# Design, synthesis, and biological evaluation of novel substituted thiourea derivatives as potential anticancer agents for NSCLC by blocking K-Ras protein-effectors interactions

**DOI:** 10.1080/14756366.2019.1702653

**Published:** 2019-12-18

**Authors:** Yuan Zhang, Xin Meng, Haikang Tang, Minghui Cheng, Fujun Yang, Wenqing Xu

**Affiliations:** Tianjin Key Laboratory of Radiation Medicine and Molecular Nuclear Medicine, Institute of Radiation Medicine, Chinese Academy of Medical Sciences and Peking Union Medical College, Tianjin, China

**Keywords:** K-Ras mutations, malignant carcinoma, anticancer drug, non-small cell lung cancer, A459 cell

## Abstract

Mutation of the proto-oncogene K-Ras is one of the most common molecular mechanisms in non-small cell lung cancer. Many drugs for treating lung cancer have been developed, however, due to clinical observed K-Ras mutations, corresponding chemotherapy and targeted therapy for such mutation are not efficient enough. In this study, on the basis of the crystal structure of K-Ras, 21 analogues (TKR01–TKR21) containing urea or thiourea were rationally designed, which can effectively inhibit the lung cancer cell A549 growth. The designing of these compounds was based on the structure of K-Ras protein, and the related groups were replaced by bioisosteres to improve the affinity and selectivity. Biological testing revealed that compound TKR15 could significantly inhibit the proliferation of A549 cell with IC_50_ of 0.21 µM. Docking analysis showed that the TKR15 can effectively bind to the hydrophobic cavity and form a hydrogen bond with the Glu37. In addition, through flow apoptosis assay and immunofluorescence staining assay, it confirmed that this compound can inhibit A549 cell proliferation with the mechanism of blocking K-Ras^G12V^ protein and effector proteins interactions through the apoptotic pathway. In conclusion, our studies in finding novel potent compound (TKR15) with confirmed mechanism showed great potential for further optimisation and other medicinal chemistry relevant studies.

## Introduction

Ras proteins are small and membrane-bound guanine nucleotide-binding proteins; they serve as molecular switches between active guanine triphosphate (GTP)-bound and inactive guanine diphosphate-bound conformations. The Ras proteins usually consist of 188 or 189 amino acids. The 1–165 amino acids of each subtype have around 92–98% identical sequences, they are highly conserved during evolution and called G regions, which are responsible for the passage of guanine nucleotides^1^. The remaining amino acids vary widely and are known as the hyper variable region, and the Ras proteins are located in the cytoplasmic membrane binding to the lipid^1^. Ras proteins have a crucial role in the regulation of cell proliferation, differentiation and survival through some critical pathways, such as the Raf–MAPK/ERK kinase–extracellular signal-regulated kinase (Raf–MEK–ERK), phosphoinositide 3-kinase–AKT–mechanistic target of rapamycin (PI3K–AKT–mTOR), and Ral guanine nucleotide-dissociation stimulator–RAL (RalGDS–Ral) pathways.

Ras proto-oncogene and proteins dysfunction exist in a variety of malignant tumours. The Ras proto-oncogene encodes four subtypes of Ras proteins including H-Ras, N-Ras, K-Ras4A, and K-Ras4B, of which K-Ras4A and K-Ras4B are different splice variants of the K-Ras gene^2^. And because of different Ras proto-oncogene subtypes presenting in different cells, Ras protein mutations will also lead to different tumours^3^. H-Ras protein mutations mainly occur in the cancer of brain and neck; K-Ras protein mutations mainly occur in pancreatic cancer, colon cancer and lung cancer; N-Ras mutations are mainly found in skin cancer and blood diseases^3–5^. The Ras protein mutations are often key mutations, with approximately 99% of the mutations at positions 12 and 13 of glycine and 61 of glutamine^4^. These mutations will result in the decrease of the catalytic hydrolysis activity of GAP, a hindrance of the GAP-mediated GTP hydrolysis process, and finally a significant increase in the level of activation of Ras-GTP binding. Consequently, these processes sustained of the downstream effector proteins activation^6–8^, which finally contribute to the forming of malignant tumour.

K-Ras protein, like other members of the Ras proteins family, is a switch comprising of GTP-binding complex which regulates signalling pathways related to cell growth, differentiation, and apoptosis by interacting with multiple effector proteins^9^. Currently, there are not existing therapies specifically for targeting mutant K-Ras for the treatment of non-small cell lung cancer (NSCLC)^10^^,^^11^. But potential alternative therapeutic strategies in this direction are always being pursued, with particular efforts directed towards the inhibition of interactions between K-Ras protein and downstream effector proteins^12^^,^^13^. The development of small molecule inhibitors targeting mutant K-Ras and inhibiting K-Ras-effector proteins interactions in NSCLC will provide very important preclinical research information.

**Figure 1.**
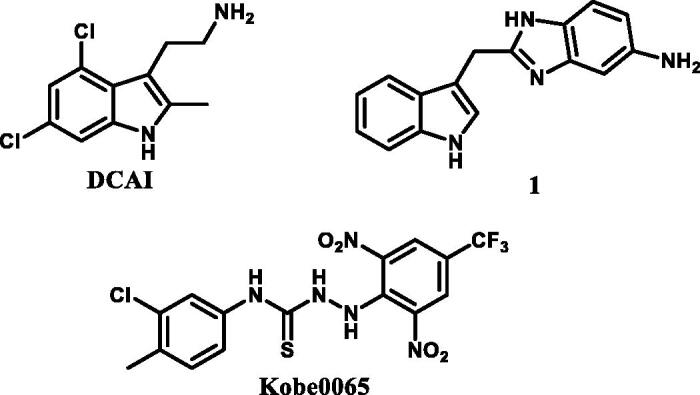
Three reported compounds targeting K-Ras^G12V^ protein.

In 2012, Stephen W. Fesik’s group^14^ and Guowei Fang’s group^15^, through fragment-based virtual screening and high-resolution crystallographic studies, respectively, found DCAI and compound 1 (Figure 1) which could efficiently bind to K-Ras protein. In 2013, Tohru Kataoka’s group^16^ discovered bioactive small compound Kobe0065 (Figure 1), through virtual screening and bioactivity evaluation, that effectively binded K-Ras protein and significantly inhibited the K-Ras protein-effector proteins interactions. By the analysis of the binding cavity, it was found that although the protein crystal models used in molecular docking and the novel binding fragments were different, but the binding sites were basically comprised of the same amino acid residues containing Lys5, Glu37, Aap54, Leu56, Met67, Gln70, Tyr71, and Thr74. Meanwhile, the structure-activity relationship (SAR) of compound Kobe0065 was not discussed by Tohru Kataoka. In addition, the compound Kobe0065 was just used to inhibit the growth and proliferation of pancreatic carcinoma and colorectal carcinoma, with a slightly higher IC_50_ value compared with marked antitumor agent Sorafenib, both of which leave space to further optimise this small molecule.

Encouraged by these results, in this study we designed and synthesised a series of compounds with structure of urea or thiourea on the basis of the lead compound Kobe0065, and examined their cytotoxic effects on NSCLC A549 cell, in order to find small molecule that could target K-Ras protein, block K-Ras-effector proteins interactions and significantly inhibit the growth of NSCLC. As a result, the structure of compound Kobe0065 was optimised and the SAR was discussed. For the most desirable compound TKR15 with IC_50_ of 0.21 µM, we have carried out molecular docking and cell-based experiments to gain the mechanism of function. The results of this study will help to develop and design future new generations of small molecule compounds that could further improve on targeting mutant K-Ras protein and cytotoxic efficacy for NSCLC.

## Materials and methods

### Materials and instruments

The commercial reagents and materials were purchased from commercial suppliers such as Sigma-Aldrich (Shanghai, China) or TCI (Shanghai, China), and used without purification. K-Ras Antibody (Rabbit Polyclonal) was purchased from Proteintech Group Inc (Wuhan, China). Raf-1 Antibody (Rabbit Polyclonal), the anti-mouse IgG and anti-rabbit secondary antibodies raised from goat were obtained from Abcam (Shanghai, China). Sorafenib Tosylate was purchased from MCE (Shanghai, China).

All melting points were measured by a Melting Point YRT-3apparatus (Tianjin precision apparatus factory, China) and were corrected. NMR spectra were performed using 300 MHz spectrometers (Bruker, USA) with TMS as an internal standard. High resolution mass spectra were determined by Thermo Scientific Exactive Plus mass spectrometry with ESI method (Thermo, USA). The purity of all these synthesised compounds was determined by HPLC analysis (Waters, USA). TLC analysis was carried out on silica gel plates GF254 (Yantai chemical research institute, China). Column chromatography was performed on silica gel (200–300 mush; Qingdao Marine Chemical Inc.).

### General procedures for synthesis

Compounds TKR01-TKR21 were synthesised as described methods in previous literatures (all the 1H NMR Spectra of Compounds TKR01-21 in Supporting Information)^17–22^.

#### Synthetic routes of target compounds TKR01–TKR07 were outlined in Scheme 1

Compound A (1.0 equiv.) dissolved in anhydrous methanol (MeOH) was added to sodium methylate (1.0 equiv.) in small portions under magnetic stirring, and continued stirring at room temperature for 1–2 h. The mixture quickly turned yellow and then gradually changed into red. After the complete depletion of A, the reaction mixture was extracted with dichloromethane and washed with distilled water to obtain the product B without further purification.

**Scheme 1.**
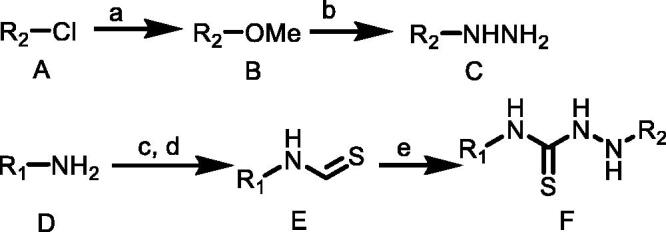
(a) MeONa, MeOH, rt; (b) NH_2_NH_2_, MeOH; (c) CS_2_, TEA, THF, rt; (d) Boc_2_O, DMAP, 5 °C to rt; (e) C, CH_3_CN, TEA, rt. R_1_ = (*o*-tolyl)/(*p*-tolyl)/(3-chlorophenyl)/(2-chlorophenyl)/(4-(trifluoromethyl)phenyl)/(naphthalen-1-yl)/(3,5-dimethylphenyl); R_2_ = (2,6-dinitro-4-(trifluoromethyl)phenyl).

A solution of hydrazine monohydrate (1.1 equiv.) in anhydrous ethanol was added dropwise to a solution of B (1.0 equiv.) in anhydrous ethanol under an argon atmosphere. The reaction mixture was stirred at 0 °C for 0.5 h and then stirred at room temperature, monitored by TLC. After the complete depletion of B, the solvent was removed under vacuum, and crude product C was purified by column chromatography on silica gel in hexane/ethylacetate (4:1).

An amine D (1.0 equiv.) was dissolved in THF. While stirring, CS_2_ (10 equiv.) and triethylamine (1.1 equiv.) was added. After the complete conversion to dithiocarbamic acid salt (monitored via TLC, generally within 30–60 min), the reaction mixture was cooled in an ice bath with immediate addition of Boc_2_O (1.0 equiv.) and DMAP (0.05 equiv.) in THF. Complete consumption of dithiocarbamic acid salt proceeded within 15–60 min. Solvent and other volatiles were removed under reduced pressure yielding isothiocyanates E and used in next step without further purification.

To a stirred solution of substituted hydrazine C (1.1 equiv.) in enough anhydrous acetonitrile, then triethylamine (3.0 equiv.) was added to the reaction mixture and appropriately substituted isothiocyanate E (1.0 equiv.) was added at room temperature. There sultant mixture was heated at reflux with magnetic stirring for 3 h or stirred at room temperature for 4 h. Precipitate was formed immediately. The mixture was filtered, and the precipitate was washed with acetonitrile or dichloromethane three times to give the desired product thioureas F.

#### *2–(2,6-Dinitro-4-(trifluoromethyl)phenyl)-*N*-(*o*-tolyl)hydrazine-1-carbothioamide (TKR01)*

This compound was obtained as claybank solid in 39% yield; Melting point: 181–182 °C.^1^H NMR (300 MHz, CDCl_3_) δ 9.67 (s, 1H), 8.39 (s, 2H), 7.71 (s, 1H), 7.37 (d, *J* = 2.8 Hz, 3H), 7.20 (s, 2H), 2.30 (s, 3H).^13^C NMR (75 MHz, DMSO) δ 137.96, 130.62, 127.29, 126.35, 124.89, 121.29, 17.99. HRMS (ESI): calculated for C_15_H_12_F_3_N_5_O_4_S [M + H]^+^: 416.0596; found:416.0628.

#### *2–(2,6-Dinitro-4-(trifluoromethyl)phenyl)-*N*-(*p*-tolyl)hydrazine-1-carbothioamide (TKR02)*

This compound was obtained as claybank solid in 38% yield; Melting point: 179–180 °C. ^1^H NMR (300 MHz, CDCl_3_) δ 9.67 (s, 1H), 8.42 (s, 2H), 7.90 (s, 1H), 7.33 (t, *J* = 7.2 Hz, 3H), 7.12 (d, *J* = 8.2 Hz, 2H), 2.42 (d, *J* = 8.3 Hz, 3H).^13^C NMR (75 MHz, DMSO) δ 138.06, 136.72, 135.16, 129.22, 127.44, 124.86, 121.26, 117.66. HRMS (ESI): calculated for C_15_H_12_F_3_N_5_O_4_S [M + H]^+^: 416.0596; found:416.0628.

#### N*-(3-Chlorophenyl)-2–(2,6-dinitro-4-(trifluoromethyl)phenyl)hydrazine-1-carbothioamide (TKR03)*

This compound was obtained as yellow solid in 36% yield; Melting point: 174–175 °C. ^1^H NMR (300 MHz, CDCl_3_) δ 9.70 (s, 1H), 8.42 (s, 2H), 8.05 (s, 1H), 7.55 – 7.30 (m, 4H), 7.21 (d, *J* = 7.7 Hz, 1H).^13^C NMR (75 MHz, DMSO) δ 148.46 – 145.99, 140.90, 138.02, 132.80, 130.33, 127.51, 125.17, 121.23 (s), 119.08 – 115.80. HRMS (ESI): calculated for C_14_H_9_ClF_3_N_5_O_4_S [M + H]^+^: 436.0049; found:436.0083.

#### N*-(2-Chlorophenyl)-2–(2,6-dinitro-4-(trifluoromethyl)phenyl)hydrazine-1-carbothioamide (TKR04)*

This compound was obtained as yellow solid in 37% yield; Melting point: 171–172 °C. ^1^H NMR (300 MHz, CDCl3) δ 9.93 – 9.62 (m, 1H), 8.64 – 8.21 (m, 2H), 7.97 (s, 1H), 7.62 – 7.32 (m, 5H).^13^C NMR (75 MHz, DMSO) δ 138.28, 129.85, 127.56, 124.85, 121.25. HRMS (ESI): calculated for C_14_H_9_ClF_3_N_5_O_4_S [M + H]^+^: 436.0049; found: 436.0074.

#### *2–(2,6-Dinitro-4-(trifluoromethyl)phenyl)-*N*-(4-(trifluoromethyl)phenyl)hydrazine-1-carbothioamide (TKR05)*

This compound was obtained as yellow solid in 41% yield; Melting point: 171–173 °C. ^1^H NMR (300 MHz, CDCl_3_) δ 9.69 (s, 1H), 8.41 (s, 2H), 8.21 (s, 1H), 7.75 (d, *J* = 8.4 Hz, 2H), 7.58 (s, 1H), 7.47 (d, *J* = 8.3 Hz, 2H).^13^C NMR (75 MHz, DMSO) δ 143.21, 138.10, 127.54, 126.19, 124.83, 122.95, 121.23. HRMS (ESI): calculated for C_15_H_9_F_6_N_5_O_4_S [M + H]^+^: 470.0313; found:470.0352.

#### *2–(2,6-Dinitro-4-(trifluoromethyl)phenyl)-*N*-(naphthalen-1-yl)hydrazine-1-carbothioamide (TKR06)*

This compound was obtained as light yellow solid in 43% yield; Melting point: 204–205 °C. ^1^H NMR (300 MHz, CDCl_3_) δ 9.67 (s, 1H), 8.38 (s, 2H), 8.05 – 7.85 (m, 4H), 7.75 – 7.53 (m, 3H), 7.45 (d, *J* = 7.2 Hz, 1H).^13^C NMR (75 MHz, DMSO) δ 134.18, 128.34, 127.47, 126.45, 125.91, 124.92, 121.32. HRMS (ESI): calculated for C_18_H_12_F_3_N_5_O_4_S [M + H]^+^: 452.0596; found:452.0628.

#### N*-(3,5-Dimethylphenyl)-2–(2,6-dinitro-4-(trifluoromethyl)phenyl)hydrazine-1-carbothioamide (TKR07)*

This compound was obtained as light yellow solid in 40% yield; Melting point: 194–195 °C. ^1^H NMR (300 MHz, CDCl_3_) δ 9.70 (s, 1H), 8.43 (s, 2H), 7.85 (s, 1H), 7.37 (s, 1H), 7.07 (s, 1H), 6.82 (s, 2H), 2.42 (s, 5H).^13^C NMR (75 MHz, DMSO) δ 138.42, 127.37, 124.86, 121.26, 117.67. HRMS (ESI): calculated for C_16_H_14_F_3_N_5_O_4_S [M + H]^+^: 430.0752; found:430.0777.

#### Synthetic routes of target compounds TKR08-TKR09 were outlined in Scheme 2

A solution of amine D (1.0 equiv.) dissolved in dichloromethane was added dropwise to a solution of triphosgene (1.5 equiv.) in dry dichloromethane with continuous stirring under nitrogen atmosphere in an ice bath. Catalytic amount of triethylamine in dichloromethane was added dropwise to the mixture. The reaction mixture was stirred for 0.5 h at room temperature and for another 3 h at 50–60 °C. The solvent was removed under vacuum to obtain product substituted isocyanates G.

**Scheme 2.**
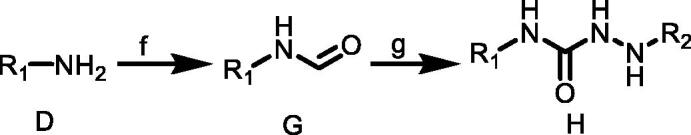
(f) BTC, TEA, EA, 50 °C; (g) C, CH_3_CN, TEA, rt. R_1_ = 4-(trifluoromethyl)phenyl)/(3-chloro-4-methylphenyl); R_2_ = (2,6-dinitro-4-(trifluoromethyl)phenyl).

An appropriate substituted hydrazine C (1.1 equiv.) was dissolved in dichloromethane, and then triethylamine (3.0 equiv.) was added to the reaction mixture. A solution of substituted isocyanate G (1.0 equiv.) in dichloromethane was added dropwise under stirring. The reaction mixture was stirred at room temperature for 2 h. The solvent was removed under reduced pressure and the residue was recrystallised from EtOH or hexane to obtain pure product ureas H.

#### N*-(3-Chloro-4-methylphenyl)-2–(2,6-dinitro-4-(trifluoromethyl)phenyl)hydrazine-1-carboxamide (TKR08)*

This compound was obtained as kelly solid in 43% yield; Melting point: 204–206 °C.^1^H NMR (300 MHz, DMSO) δ 9.80 (s, 1H), 9.01 (s, 1H), 8.51 (d, *J* = 22.1 Hz, 3H), 7.65 (d, *J* = 19.6 Hz, 1H), 7.23 (s, 3H), 2.25 (s, 4H). ^13 ^C NMR (75 MHz, DMSO) δ 155.90, 141.96, 140.11, 137.30, 133.54, 131.55, 129.14, 127.57, 124.85, 121.25, 118.33. HRMS (ESI): calculated for C_15_H_11_ClF_3_N_5_O_5_ [M + H]^+^: 434.0434; found:434.0478.

#### *2–(2,6-Dinitro-4-(trifluoromethyl)phenyl)-*N*-(4-(trifluoromethyl)phenyl)hydrazine-1-carboxamide (TKR09)*

This compound was obtained as yellow solid in 41% yield; Melting point: 202–203 °C. ^1^H NMR (300 MHz, DMSO) δ 9.83 (s, 1H), 9.33 (s, 1H), 8.58 (s, 1H), 8.56 (s, 2H), 7.64 (d, *J* = 2.4 Hz, 5H).^13^C NMR (75 MHz, DMSO) δ 155.82, 143.26, 138.03, 127.64, 126.53, 124.83, 123.13, 121.23, 118.74, 113.45. HRMS (ESI): calculated for C_15_H_9_F_6_N_5_O_5_ [M + H]^+^: 454.0541; found:454.0586.

#### Synthetic routes of target compounds TKR10–TKR18 were outlined in Scheme 3

To a solution of amine I (1.1 equiv.) in anhydrous acetonitrile was added triethylamine (3.0 equiv.), and then the resulted solution was added isocyanate G or isothiocyanate E (1.0 equiv.). The reaction mixture was then stirred until complete conversion of the starting material monitored by TLC. The solvent was removed under reduced pressure and the residue was recrystallised from EtOH or hexane to get the desired ureas J or thioureas K.

**Scheme 3.**
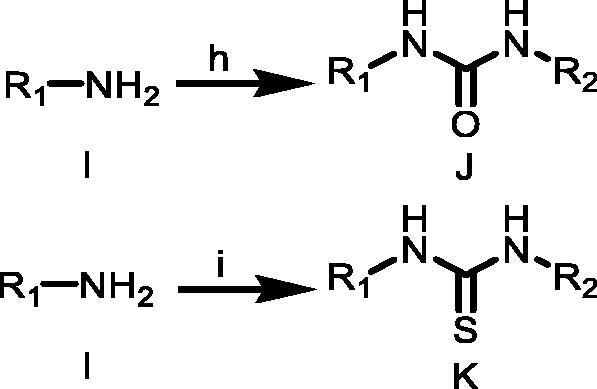
(h) G, CH3CN, TEA, rt; (i) E, CH3CN, TEA, rt. R_1_ = 4-(trifluoromethyl)phenyl)/(3-chloro-4-methylphenyl); R_2_ = mesitylene/4-(trifluoromethyl)phenyl)/(1,1′-biphenyl)/(3s,5s,7s)-adamantan-1-yl)/(4-phenoxyphenyl).

#### 1–(3-Chloro-4-methylphenyl)-3-mesitylurea (TKR10)

This compound was obtained as light yellow solid in 45% yield; Melting point: 261–262 °C. ^1^H NMR (300 MHz, DMSO) δ 8.78 (s, 1H), 7.69 (d, *J* = 1.8 Hz, 1H), 7.66 (s, 1H), 7.27 – 7.12 (m, 2H), 6.88 (s, 2H), 2.24 (d, *J* = 4.4 Hz, 6H), 2.15 (s, 6H).^13^C NMR (75 MHz, DMSO) δ 153.72, 140.05, 135.83, 135.49, 133.53, 132.95, 131.48, 128.79, 128.08, 118.37, 117.14. HRMS (ESI): calculated for C_17_H_19_ClN_2_O [M + H]^+^: 303.1219; found: 303.1262.

#### 1–(3-Chloro-4-methylphenyl)-3–(4-(trifluoromethyl)phenyl)urea (TKR11)

This compound was obtained as light yellow solid in 46% yield; Melting point: 235–236 °C. ^1^H NMR (300 MHz, DMSO) δ 9.15 (s, 1H), 8.91 (s, 1H), 7.69 (t, *J* = 2.2 Hz, 1H), 7.64 (s, 4H), 7.22 (dt, *J* = 8.3, 5.2 Hz, 2H), 2.26 (s, 4H). ^13 ^C NMR (75 MHz, DMSO) δ 152.66, 143.77, 139.08, 133.64, 131.55, 129.10, 126.89, 125.99, 123.19, 122.64, 122.21, 118.89, 118.40, 117.56. HRMS (ESI): calculated for C_15_H_12_ClF_3_N_2_O [M + H]^+^: 329.0624; found: 329.0676.

#### 1-Mesityl-3–(4-(trifluoromethyl)phenyl)urea (TKR12)

This compound was obtained as light yellow solid in 44% yield; Melting point: 207–209 °C. ^1^H NMR (300 MHz, DMSO) δ 9.15 (s, 1H), 7.78 (s, 1H), 7.63 (q, *J* = 8.9 Hz, 5H), 6.90 (s, 2H), 2.24 (s, 4H), 2.16 (s, 7H).^13^C NMR (75 MHz, DMSO) δ 153.51, 144.58, 135.73, 132.78, 128.80, 126.38, 118.01. HRMS (ESI): calculated for C_17_H_17_F_3_N_2_O [M + H]^+^: 323.1327; found: 323.1367.

#### 1,3-bis(4-(Trifluoromethyl)phenyl)urea (TKR13)

This compound was obtained as light grey solid in 49% yield; Melting point: 237–238 °C. ^1^H NMR (300 MHz, DMSO) δ 9.26 (s, 2H), 7.72 – 7.63 (m, 8H).^13^C NMR (75 MHz, DMSO) δ 152.55, 143.54, 126.73 – 125.87, 123.13, 122.92, 122.50, 118.51. HRMS (ESI): calculated for C_15_H_10_F_6_N_2_O [M + H]^+^: 349.0731; found: 349.0779.

#### 1-Mesityl-3–(4-(trifluoromethyl)phenyl)thiourea (TKR14)

This compound was obtained as grey solid in 45% yield; Melting point: 204–205 °C. ^1^H NMR (300 MHz, CDCl3) δ 7.70 (s, 1H), 7.58 (q, *J* = 8.4 Hz, 4H), 7.10 (s, 1H), 6.99 (d, *J* = 13.4 Hz, 2H), 2.31 (d, *J* = 6.5 Hz, 9H).^13^C NMR (75 MHz, DMSO) δ 180.96, 143.99, 136.38, 128.81, 126.12, 122.47. HRMS (ESI): calculated for C_17_H_17_F_3_N_2_S [M + H]^+^: 339.1098; found: 339.1141.

#### 1,3-bis(4-(Trifluoromethyl)phenyl)thiourea (TKR15)

This compound was obtained as grey solid in 49% yield; Melting point: 179–181 °C. ^1^H NMR (300 MHz, CDCl3) δ 8.15 (s, 1H), 7.70 (d, *J* = 8.5 Hz, 3H), 7.56 (d, *J* = 8.4 Hz, 3H).^13^C NMR (75 MHz, DMSO) δ 180.17 (s), 143.54 (s), 126.29 – 125.92 (m), 123.49 (s). HRMS (ESI): calculated for C_15_H_10_F_6_N_2_S [M + H]^+^: 365.0502; found: 365.0553.

#### 1-([1,1′-Biphenyl]-4-yl)-3–(4-(trifluoromethyl)phenyl)thiourea (TKR16)

This compound was obtained as grey solid in 45% yield; Melting point: 201–203 °C. ^1^H NMR (300 MHz, DMSO) δ 10.25 (s, 1H), 7.79 (d, *J* = 8.6 Hz, 1H), 7.66 (ddd, *J* = 19.9, 12.2, 6.8 Hz, 4H), 7.47 (dd, *J* = 10.3, 4.8 Hz, 1H), 7.39 – 7.32 (m, 1H).^13^C NMR (75 MHz, DMSO) δ 179.79, 144.01, 140.13, 139.20, 136.81, 129.40, 127.20, 126.87, 124.25, 123.14. HRMS (ESI): calculated for C_20_H_15_F_3_N_2_S [M + H]^+^: 373.0942; found: 373.0958.

#### 1-((3s,5s,7s)-Adamantan-1-yl)-3–(4-(trifluoromethyl)phenyl)thiourea (TKR17)

This compound was obtained as white solid in 46% yield; Melting point: 176–177 °C. ^1^H NMR (300 MHz, DMSO) δ 9.63 (s, 1H), 7.75 (d, *J* = 8.5 Hz, 2H), 7.62 (d, *J* = 8.7 Hz, 2H), 7.57 (s, 1H), 2.25 (d, *J* = 2.5 Hz, 6H), 2.07 (s, 3H), 1.65 (s, 6H).^13^C NMR (75 MHz, DMSO) δ 178.93, 144.08, 125.77, 122.22, 54.06, 36.44, 29.51. HRMS (ESI): calculated for C_18_H_21_F_3_N_2_S [M + H]^+^: 355.1411; found: 355.1421.

#### 1–(4-Phenoxyphenyl)-3–(4-(trifluoromethyl)phenyl)urea (TKR18)

This compound was obtained as white solid in 45% yield; Melting point: 203–204 °C. ^1^H NMR (300 MHz, DMSO) δ 9.09 (s, 1H), 8.82 (s, 1H), 7.65 (q, *J* = 9.1 Hz, 5H), 7.49 (t, *J* = 6.2 Hz, 2H), 7.38 (dd, *J* = 11.2, 4.7 Hz, 2H), 7.10 (t, *J* = 7.4 Hz, 1H), 6.99 (dd, *J* = 11.6, 6.5 Hz, 5H).^13^C NMR (75 MHz, DMSO) δ 158.07, 152.86, 151.53, 144.00, 135.74, 130.32, 126.47, 123.24, 120.73, 120.18, 118.23. HRMS (ESI): calculated for C_20_H_15_F_3_N_2_O_2_ [M + H]^+^: 373.1119; found: 373.1182.

#### Synthetic routes of target compounds TKR19–TKR21 were outlined in Scheme 4

The substituted urea or thiourea (1.0 equiv.) was dissolved in glacial acetic acid with gentle heating, and then appropriate of conc. HCl and distilled water were added. The mixture was cooled to 0–5 °C, stirring constantly. A separate solution of NaNO_2_ (1.0 equiv.) in distilled water was then prepared and cooled to 0–5 °C. With efficient stirring, this solution was then added in small aliquots to the solution of urea or thiourea, maintaining the temperature at less than 10 °C throughout. After 5 min of further stirring at or less than 5 °C, the product was vacuum filtered, washed with cold distilled water, and dried to constant weight in an oven at 50–60 °C to obtain product L.

**Scheme 4.**
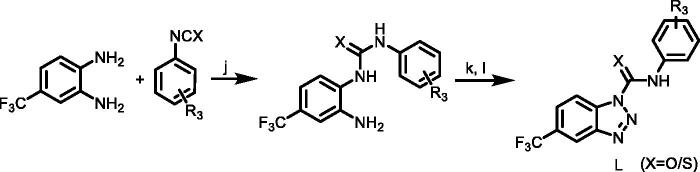
(j) DCM, TEA, 1–5 °C; (k) HCl, H_2_O, CH_3_COOH, 1–5 °C; (l) NaNO_2_/H_2_O, 1–5 °C. R_3_ = 4-(trifluoromethyl)phenyl)/(3-chloro-4-methylphenyl).

#### N*-(3-Chloro-4-methylphenyl)-5-(trifluoromethyl)-1H-benzo[d][1,2,3]triazole-1-carboxamide (TKR19)*

This compound was obtained as white solid in 39% yield; Melting point: 180–181 °C. ^1^H NMR (300 MHz, DMSO) δ 8.83 (s, 1H), 8.46 (s, 1H), 8.12 (d, *J* = 8.7 Hz, 1H), 7.77 (dd, *J* = 8.7, 1.4 Hz, 1H), 7.68 (d, *J* = 2.1 Hz, 1H), 7.20 (dt, *J* = 8.3, 5.2 Hz, 2H), 2.26 (s, 4H).^13^C NMR (75 MHz, DMSO) δ 152.78, 140.24, 139.07, 133.57, 131.47, 128.70, 122.76, 118.63, 117.33, 115.78, 115.07. HRMS (ESI): calculated for C_15_H_10_ClF_3_N_4_O [M + H]^+^: 355.0529; found:355.0541.

#### *5-(Trifluoromethyl)-*N*-(4-(trifluoromethyl)phenyl)-1H-benzo[d][1,2,3]triazole-1-carboxamide (TKR20)*

This compound was obtained as white solid in 40% yield; Melting point: 219.3 °C. ^1^H NMR (300 MHz, DMSO) δ 9.62 (d, *J* = 14.1 Hz, 1H), 8.43 (d, *J* = 32.7 Hz, 1H), 7.98 (dd, *J* = 9.7, 5.0 Hz, 1H), 7.77 – 7.60 (m, 4H), 7.49 (dd, *J* = 8.6, 1.6 Hz, 1H).^13^C NMR (75 MHz, DMSO) δ 153.44, 153.04, 143.73, 135.68, 131.15, 126.55, 118.48. HRMS (ESI): calculated for C_15_H_8_F_6_N_4_O [M + H]^+^: 375.0636; found:375.0649.

#### *5-(Trifluoromethyl)-*N*-(4-(trifluoromethyl)phenyl)-1H-benzo[d][1,2,3]triazole-1-carbothioamide (TKR21)*

This compound was obtained as white solid in 39% yield; Melting point: 135–136 °C. ^1^H NMR (300 MHz, DMSO) δ 9.62 (d, *J* = 14.1 Hz, 1H), 8.43 (d, *J* = 32.7 Hz, 1H), 7.98 (dd, *J* = 9.7, 5.0 Hz, 1H), 7.76 – 7.60 (m, 4H), 7.49 (dd, *J* = 8.6, 1.6 Hz, 1H).^13^C NMR (75 MHz, DMSO) δ 153.44, 153.04, 143.73, 135.68, 131.15, 126.66, 123.98, 118.48.13C NMR (75 MHz, DMSO) δ 178.91, 140.21, 138.92, 125.78, 122.56, 122.21, 115.76, 115.04, 54.03, 36.35, 29.43. HRMS (ESI): calculated for C_15_H_8_F_6_N_4_S [M + H]^+^: 391.0417; found:391.0407.

### Cell culture

The human NSCLC cell line A549 were purchased from Institute of Basic Medical Science of Peking Union Medical College (IBMS, PUMC, China) and maintained according to the provided protocols. Briefly, A549 cells were maintained in Dulbecco’s modified Eagle’s medium (DMEM) containing 10% foetal bovine serum (Gibbco, Gland Island, NY, USA) and 1% penicillin/streptomycin (Gibbco, Gland Island, NY, USA) at 37 °C with 5% CO_2_.

### Cell proliferation assay

To measure cell viability and proliferation, A549 cells were seeded at a final concentration of 5 × 10^3^ cells/mL, respectively, in a 96-well plate. Compounds were dissolved in DMSO, and the cells were treated with different concentrations (0.2, 1, 5, 25, and 75 µM) of compounds. Cell viability and cell proliferation were determined after 48 h of incubation, respectively. After the treatment, cell counting kit-8 reagent (MCE, Shanghai, China) was added to each well. The cells were incubated for 3 h, and the absorbance was measured at 450 nm using Spectra Max i3.

### Molecular docking

TKR15 was docked into the binding pocket of K-Ras protein (PDB code: 4DSO) using docking software Autodock Vina (Designed by Dr. Oleg Trott in the Molecular Graphics Lab at The Scripps Research Institute). All required were the structures of the molecules being docked and the specification of the search space including the binding site. Calculating grid maps and assigning atom charges was not needed. The binding pocket was searched by Autodock 4.2 (Autodock suite-4.2.6.i86Windows) according to the literature^14–16^. Water molecules were deleted and hydrogen atoms were added. The structure of compound 10 was drawn on ChemDraw and copied to Autodock 4.2. The active site of K-Ras^G12V^ was defined as the collection of amino acids enclosed within a 6.5 Å radius of compound TKR15.

### Cell apoptosis assay

In order to investigate the mechanism of compound TKR15 in inhibiting A549 growth and proliferation, the cell apoptosis assay analysed by flow cytometry was conducted. Cells were treated with 0.1% DMSO, 24 µM Sorafenib, and 1.0 µM and 2.0 µM TKR15 in 2.5% FBS-DMEM for 48 h and stained using the Alexa Fluor^®^ 488 Annexin V/Dead Cell Apoptosis Kit according to the manufacturer’s protocol (Molecular Probes). Annexin V and PI were analysed by flow cytometry. Annexin V-positive cells were counted as Apoptosis cells in total 5 × 10^4^ cells per each sample.

### Immunofluorescence staining assay

Immunofluorescence Staining Assay was conducted in the study to demonstrate the effects of blocking K-Ras-Raf-1 proteins interactions^23^. After 2 h incubation with 1% DMSO and compound TKR15 (20 µM), A549 cells were washed with PBS three times and fixated with 4% Paraformaldehyde for 5 min. After an additional three times PBS washing, cells were permeabilised with 0.1% Triton-X for 10 min and washed with PBS three times before with 1% BSA blocking buffer at a period of 30 min. The anti-K-RAS mouse monoclonal antibody was incubated in blocking buffer overnight at 4 °C. Afterwards, the cells were washed with PBS three times. A secondary antibody, goat anti-Mouse antibody, was incubated for 2 h in PBS. Then, cells were washed with PBS for three times again and incubated with the anti-Raf-1 antibody in blocking buffer overnight at 4 °C, subsequently incubated with a secondary antibody, anti-goat antibody for 2 h in PBS. The cells were washed three times with PBS and stored in PBS for microscopy.

### Statistics

All of the experiments were repeated a minimum of three times. The data are expressed as the means (SD). Statistical significance was analysed using either Unpaired-Student’s *t*-test (two-tailed) or analysis of variance test. The *p* values of less than 0.05 was considered statistically significant.

## Results and discussion

### Antiproliferative effects of compounds TKR01–TKR21 on NSCLC A549 cells

The newly synthesised urea or thiourea compounds (TKR01–TKR21) were evaluated for their antiproliferative effects using NSCLC A549 cell lines, for which these compounds were diluted to achieve five different concentrations ranging from 0.2 to 75 µM. And we choose Sorafenib as the controlled agent, which can markedly inhibit Raf-1 kinase protein. Followed by 48 h incubation with these compounds, cells were treated with cell counting kit-8reagent to measure their growth/viability (% of the untreated control) by Spectra Max i3. The 50% inhibitory concentration (IC_50_) for each derivative was calculated according to the equation of Boltzmann sigmoidal concentration-response curve using Graph Pad Prism 8, which represented the concentration that resulted in a 50% decrease in cell growth after 48 h of incubation. As shown in the Tables 1–4, several compounds out of TKR01–TKR21 can effectively inhibit A549cells growth in a dose-dependent manner. Especially for compound TKR15, it could significantly inhibit the growth of A549 cell with IC_50_ of 0.21 µM.

**Table 1.** Compounds TKR01-TKR07 and their anti-tumour activity.


Compound	R_1_	R_2_	R_3_	R_4_	IC_50_ (μM)
Sorafenib	–	–	–	–	8.50
TKR01	–CH_3_	–H	–H	–H	101.7
TKR02	–H	–H	–CH_3_	–H	>100
TKR03	–H	–Cl	–H	–H	2.72
TKR04	–Cl	–H	–H	–H	>100
TKR05	–H	–H	–CF_3_	–H	1.25
TKR06	Naphthalene	Naphthalene	–H	–H	>100
TKR07	–H	–CH_3_	–H	–CH_3_	>100

**Table 2.** Compounds TKR08-TKR09 and their anti-tumour activity.


Compound	R_4_	R_5_	IC_50_ (μM)
TKR08	–Cl	–CH_3_	125.9
TKR09	–H	–CF_3_	81.9

**Table 3.** Compounds TKR10–TKR18 and their anti-tumour activity.

**Table 4.** Compounds TKR19–TKR21 and their anti-tumour activity.


Compound	R_9_	X	R_10_	IC_50_ (μM)
TKR19	–CH_3_	O	–Cl	>100
TKR20	–CF_3_	O	–H	22.75
TKR21	–CF_3_	S	–H	18.57

Initially, as shown in Table 1, we attempted to increase the affinity binding to K-Ras protein by changing the hydrophobic groups of phenyl, such as methyl, naphthyl, and trifluoromethyl. And we finally found that only TKR03 and TKR05 can significantly inhibit A549 cells proliferation compared with Sorafenib. It demonstrates that the substitutions (2-chlorophenyl and *p*-trifluoromethyl) can markedly contribute to the hydrophobic interactions between small molecule and receptor protein in these hydrophobic groups, especially for *p*-trifluoromethyl which lead compound TKR05 inhibit A549 cell proliferation with IC_50_ of 1.25 µM. The stronger contributions of *p*-trifluoromethyl to the hydrophobic interactions may lead these observations of anti-proliferation.

As shown in Table 2, we wanted to learn about the impacts of urea and thiourea for the affinity. And we found that urea structure molecule has higher IC_50_ value compared with thiourea and Sorafenib (Table 1), in other words, the urea structure cannot effectively promote the binding between small molecule and receptor protein. This phenomenon may be owing to the different electronic effect lead by oxygen and sulphur in the structures of urea and thiourea.

In addition, we intended to shorten the linker of the two phenyls and investigate the impacts of volume of group on hydrazine. As shown in Table 3, we found that small molecule TKR15 and TKR18 can significantly inhibit A549 cells growth compared with TKR05 and Sorafenib (Table 1), with IC_50_ of 0.21 µM and 5.37 µM, respectively. This demonstrates that decreasing of hydrazine nitrogen can markedly in inhibit A549 cells and the thiourea structure is needed for the activity. At the same time, volume increasing of group on hydrazine will not contribute to inhibition, and hydrophobic group *p*-trifluoromethyl can further contribute to affinity of binding. This may be owing to that the all binding pocket need appropriately size of small molecule to interact with.

Finally, we attempted to construct a five-member cycle in the compound, because there may form hydrogen bond between nitryl and hydrazine in the interactions. But this change cannot markedly contribute to the inhibitions of compounds against A549 cells compared with TKR05 and Sorafenib (Table 1). That is to say, the rigid rings of compounds TKR19–TKR21 were not the practical conformations in the interactions with active site.

### Binding mode of small molecule TKR15 with K-Ras^G12V^ protein

Molecular docking was performed using TKR15 and the active site of K-Ras protein (PDB ID: 4DSO). In order to rationalise the biological results and to gain insight into the SAR of the most potent compound, an attempt to interpret the observed anti-proliferation activity of the tested compound TKR15 on the basis of the ligand-protein interactions was done. The anti-proliferation activity of K-Ras-effector protein interactions inhibitors depends on the ability of the compound to properly binding into the allosteric site.

**Figure 2.**
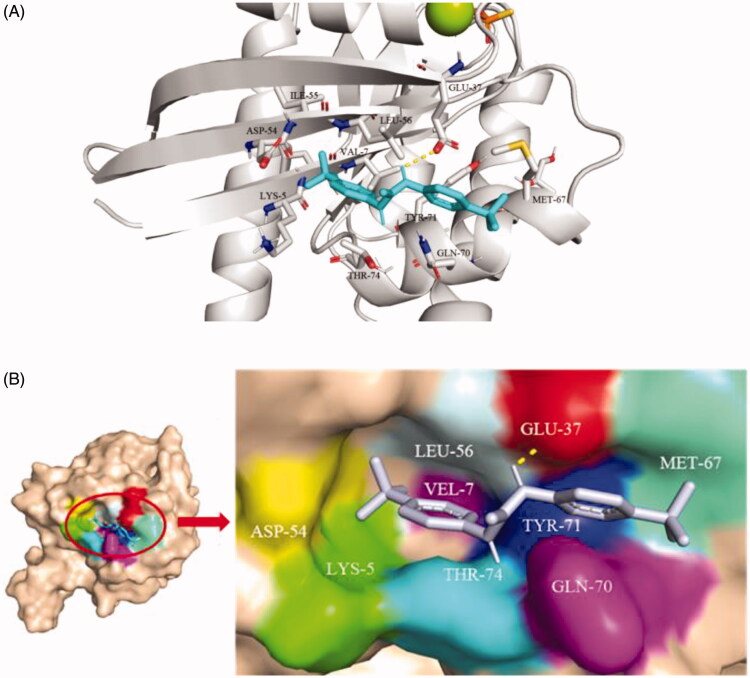
(A) Cartoon structure and (B) Surface structure of molecular docking analysis of small molecule TKR15 and K-Ras^G12V^ protein.

As shown in the Figure 2(A,B), the benzotrifluoride moiety of TKR15 was located in the active site consisting of Lys5, Glu37, Ile55, Aap54, Leu56, Met67, Gln70, Tyr71, and Thr74 residues of K-Ras^G12V^. These Lys5, Glu37, Ile55, Aap54, and Leu56 residues formed a hydrophobic surface pocket in the neighbourhood of switch, indicating that the benzotrifluoride part was inserted into the pocket through hydrophobic interaction. In addition, the residue of Glu37 can form a hydrogen bond with hydrogen of nitrogen. And the other moiety of benzotrifluoride of TKR15 was located binding site forming by Met67, Gln70, Tyr71, and Thr74. Thus, this binding mode between small molecule and receptor protein provided the binding affinity and anti-proliferation activity.

### Flow cytometric analysis of compound TKR15 induced apoptosis on A549 cells

**Figure 3.**
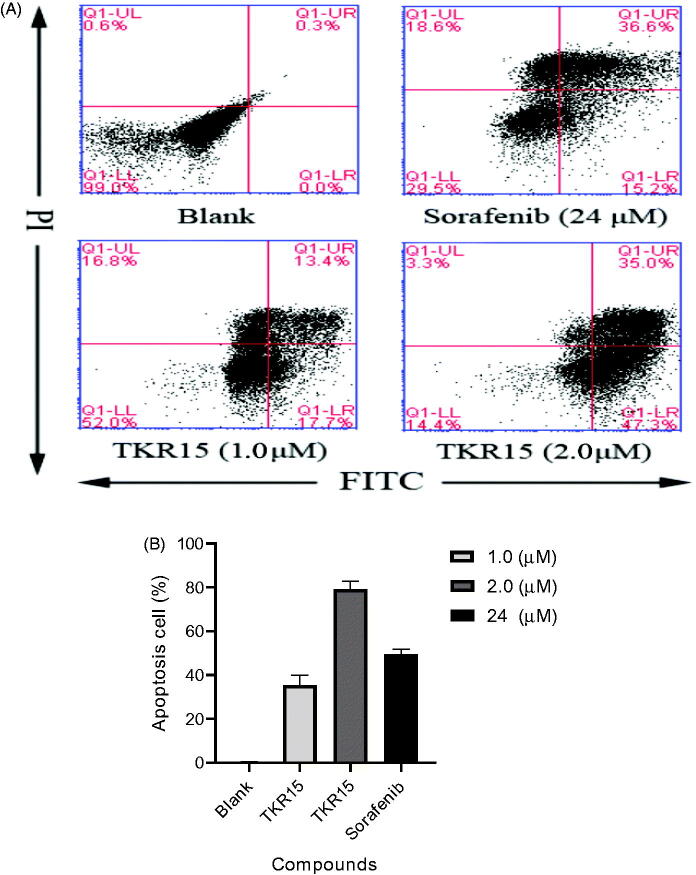
(A) Flow cytometric analysis of compound TKR-15 induced apoptosis on A549 cells; (B) Cell apoptosis amount of A549 after treating TKR15 and Sorafenib (the results were significant, *p* < 0.05, *n* = 3).

DNA mutations and protein mutations of malignant carcinoma cells can continue to multiply. The K-Ras protein mutations of NSCLC A549 also can contribute to persistent proliferation. The role of PPI inhibitors, which can target K-Ras protein and block PPI, is to inhibit the over activity of K-Ras downstream effector proteins by blocking PPI, such as Raf-1 and PI3K. And that will further induce carcinoma cells apoptosis. In other words, treatment with these anticancer agents can lead malignant carcinoma cells apoptosis by blocking K-Ras PPI. In this study, A549 cells were treated with compound TKR15 at 1.0 and 2.0 µM and controlled agent Sorafenib at 24 µM. As shown in Figure 3(A,B), compound TKR15 resulted in apoptosis of A549 cells (31.1% and 82.3%, respectively; Figure 3(A)), compared with blank group, and the controlled group in a dose-dependent manner. Moreover, when improved the concentration of TKR15, the proportion of apoptosis cells was significantly increasing, while Sorafenib resulted in apoptosis of A549 cells around 50% (Figure 3(B)). These results demonstrate that compound TKR15 antitumor activity is better than Sorafenib by targeting K-Ras protein and blocking PPI.

### Effects of compound TKR15Blocking K-Ras protein-Raf-1 protein interaction

**Figure 4.**
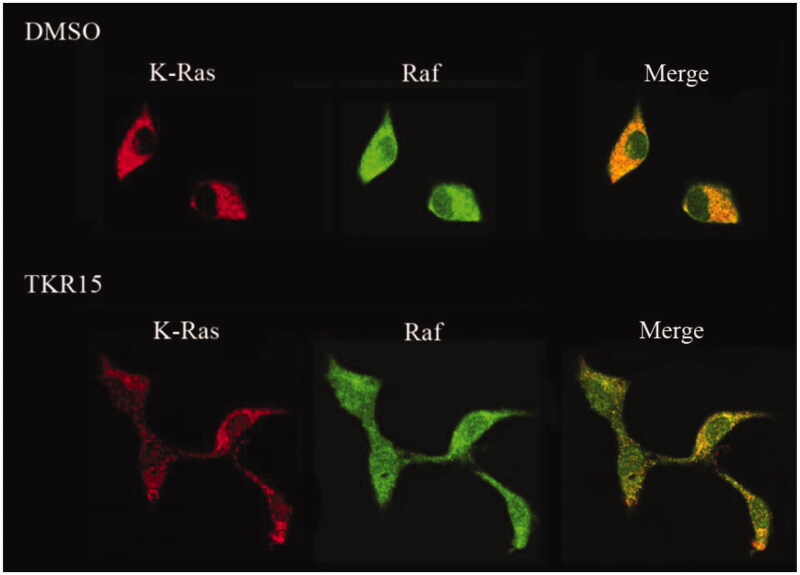
Immunostaining of A549 after treatment with compound TKR15 for 2 h (20 μM). The vehicle control for the above experiments was 0.1% DMSO.

If compound can significantly block K-Ras-effector proteins interactions, K-Ras protein and effector proteins cannot be close to each other in the space. In order to directly observe the effects of blocking PPI of compound TKR15, we conducted an immunofluorescence staining experiment. In this experiment, after treatment compound TKR15, the K-Ras protein was stained into red colour, and Raf-1 protein was stained green colour. And then, these cells were observed under laser scanning confocal microscope, and we obviously found that there were several red colour dots in the TKR15 group compared with blank (DMSO) group after being merged (Figure 4). This result can directly demonstrate that compound TKR15 can significantly target K-Ras protein and inhibit K-Ras protein-Raf-1 protein interaction.

## Conclusion

K-Ras protein is an attractive and effective therapeutic target because of such crucial role in the cell signalling paths. However, most of the attempts to develop targeting at K-Ras protein inhibitors have faced huge challenges due to their low druggability. At the same time, protein-protein interactions inhibitors offer another strategy to study targeting K-Ras protein with the role of inducing apoptosis of carcinoma cell.

In this study, we have designed a series of compounds on the basis of Kobe0065, one of Kobe0065-family compounds with confirmed mechanism action against K-Ras which further showed antitumor activity by blocking the Ras-effctor interaction. Under these backgrounds, more than 20 novel inhibitors were designed, synthesised and biologically tested using the lead compound (Kobe0065) as template. Among these inhibitors, a promising analogue TKR15 could inhibit A549 cell growth in a dose dependent manner. Moreover, by flow cytometry assay, compound TKR15 significantly induced the apoptosis of A549 cells with the percentage of apoptosis cells ranging from around 30% to around 80% compared with the control group. Immunofluorescence staining assay demonstrates that TKR15 can significantly inhibit K-Ras-effector proteins interactions. And molecular docking demonstrates that small molecule TKR15 bind to the active pocket through the strong hydrophobic interactions between benzotrifluoride moiety and binding site forming by Gln70, Tyr71, and Thr74 and hydrogen bond between Glu37 and hydrogen of nitrogen.

Taken together, compound TKR15 performed stronger anticancer activity towards A549 cells. It inhibited cells proliferation and induced apoptosis of A549 cells via targeting the K-Ras^G12V^ protein. In conclusion, urea and thiourea derivatives should be served as a promising antitumor leads with good promising to be further exploited.

## Supplementary Material

Supplemental Material
