# Supplementary material for: Design, synthesis, and biological evaluation of novel substituted thiourea derivatives as potential anticancer agents for NSCLC by blocking K-Ras protein-effectors interactions
Source: J Enzyme Inhib Med Chem. 2019 Dec 18;35(1):344–53. doi: 10.1080/14756366.2019.1702653 (PMC6968486; doi:10.1080/14756366.2019.1702653)
Supplement: Supplemental Material [file IENZ_A_1702653_SM5323.pdf]

Supporting Information

(Copies of the ^{1}H NMR Spectra)

The ^{1}H NMR data of compounds TKR01-TKR21 was measured by 300M Bruker nuclear magnetic spectroscope.

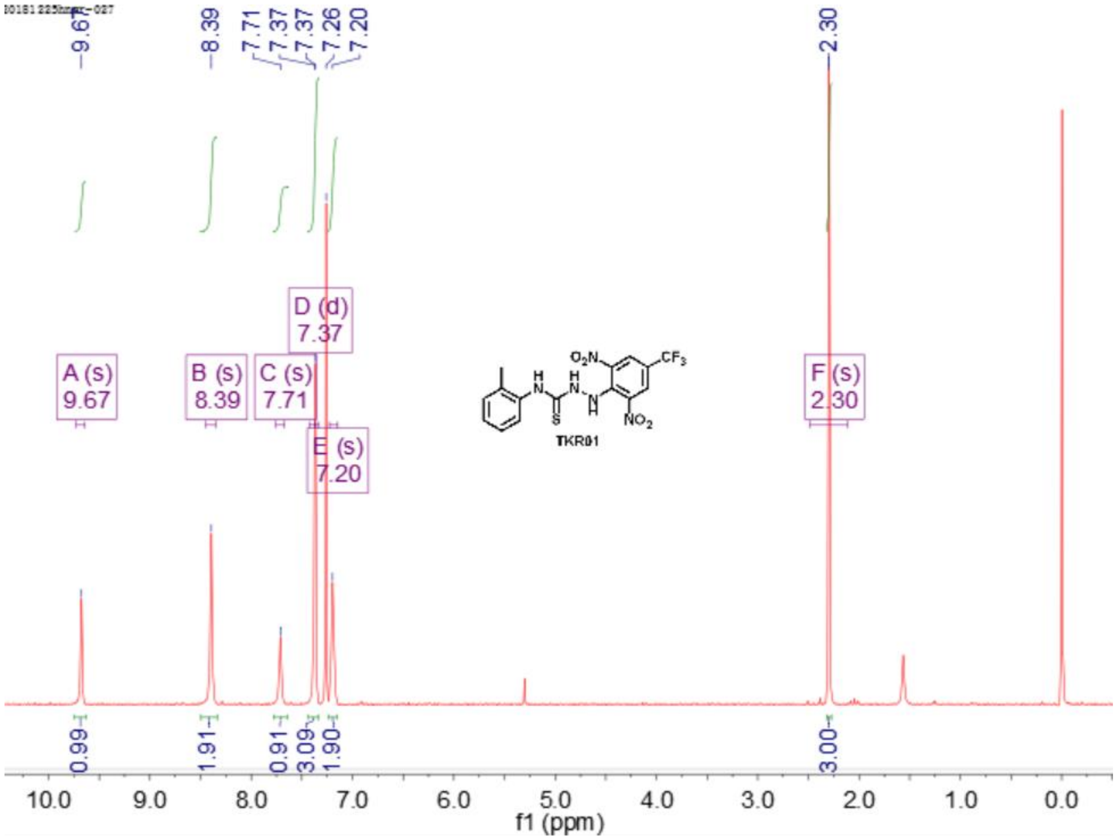

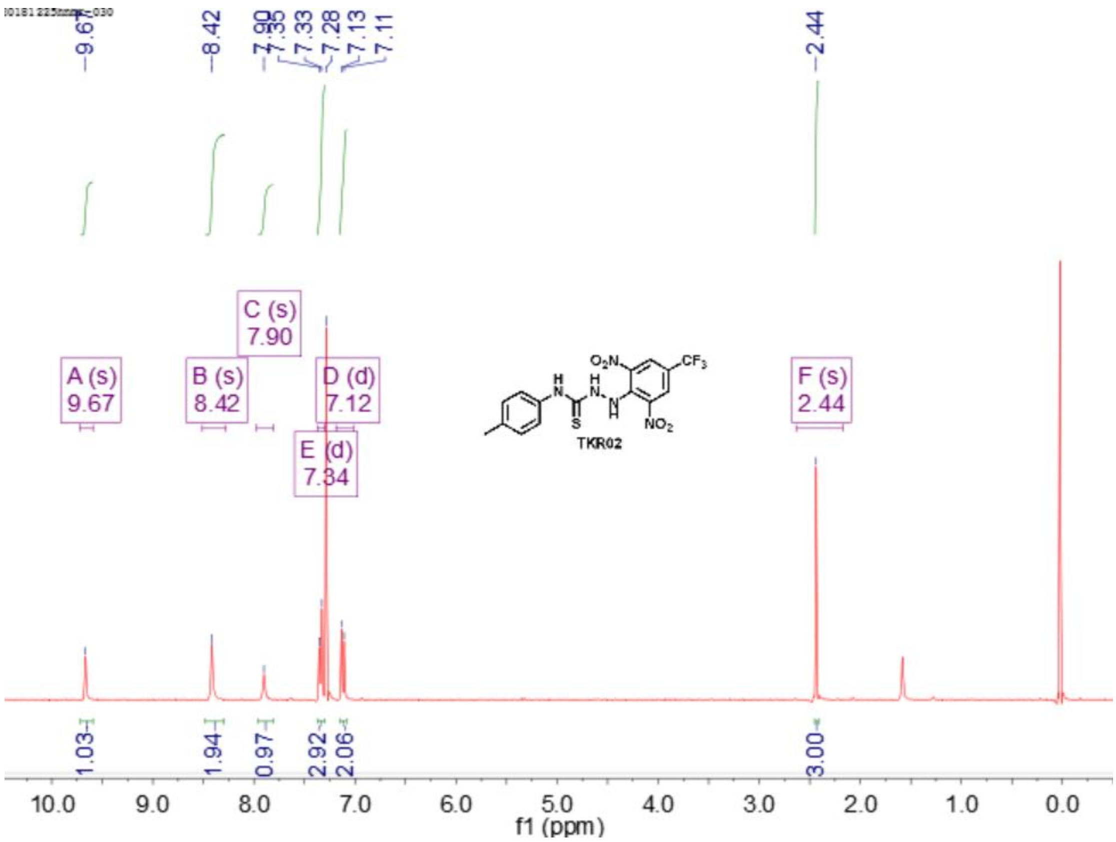
9.67
8.42
7.90
7.35
7.33
7.28
7.13
7.11
2.44
A (s) 9.67
B (s) 8.42
C (s) 7.90
D (d) 7.12
E (d) 7.34
F (s) 2.44
TKR02
1.03
1.94
0.97
2.92
2.06
3.00
f1 (ppm)

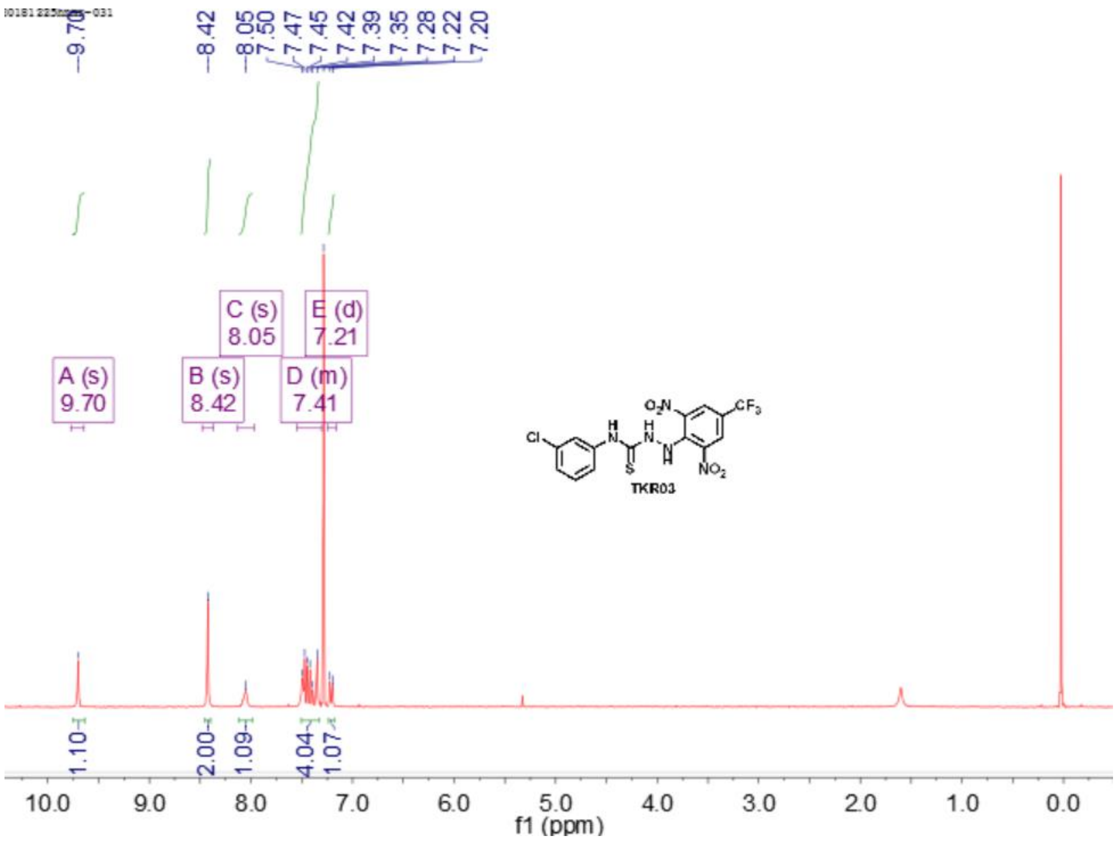
9.70
8.42
8.05
7.50
7.47
7.45
7.42
7.39
7.35
7.28
7.22
7.20
A (s) 9.70
B (s) 8.42
C (s) 8.05
D (m) 7.41
E (d) 7.21
TKR03
1.10
2.00
1.09
4.04
1.07
f1 (ppm)

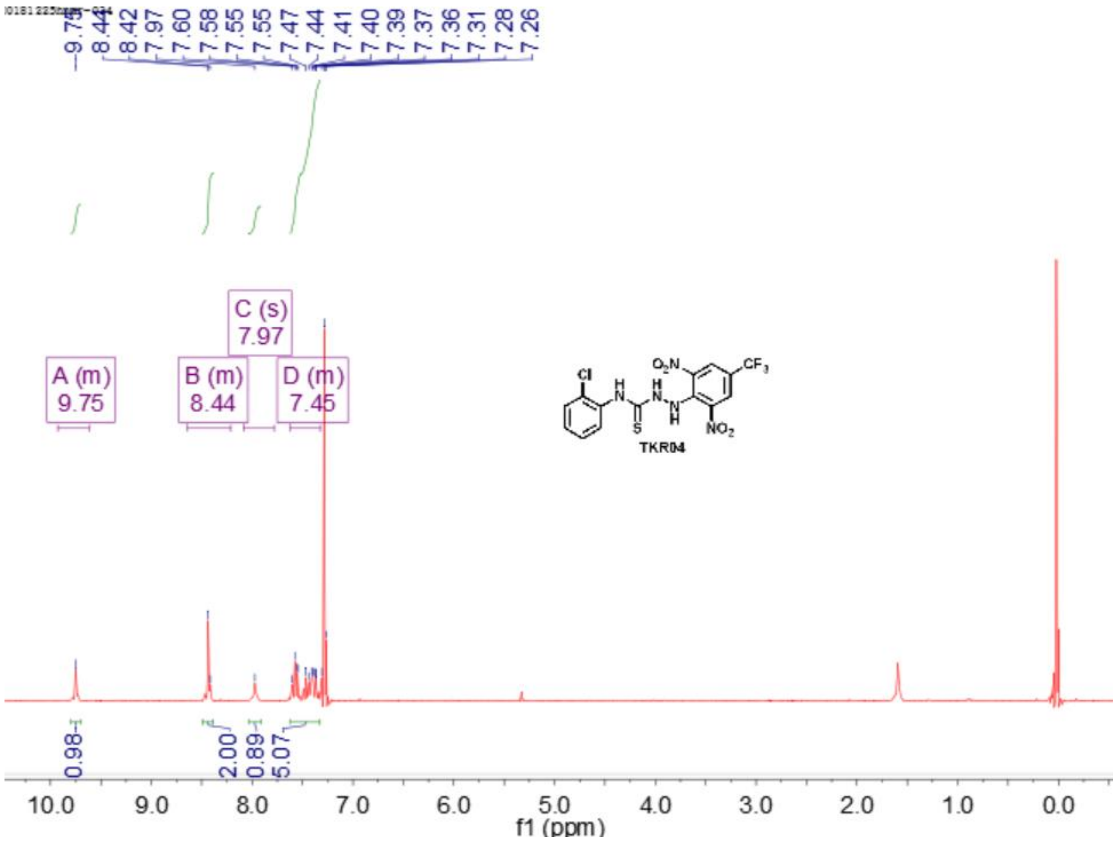
9.74 8.44 8.42 7.97 7.60 7.58 7.55 7.55 7.47 7.44 7.41 7.40 7.39 7.37 7.36 7.31 7.28 7.26
A (m) 9.75
B (m) 8.44
C (s) 7.97
D (m) 7.45
TKR04
0.98 2.00 0.89 5.07
10.0 9.0 8.0 7.0 6.0 5.0 4.0 3.0 2.0 1.0 0.0
f1 (ppm)

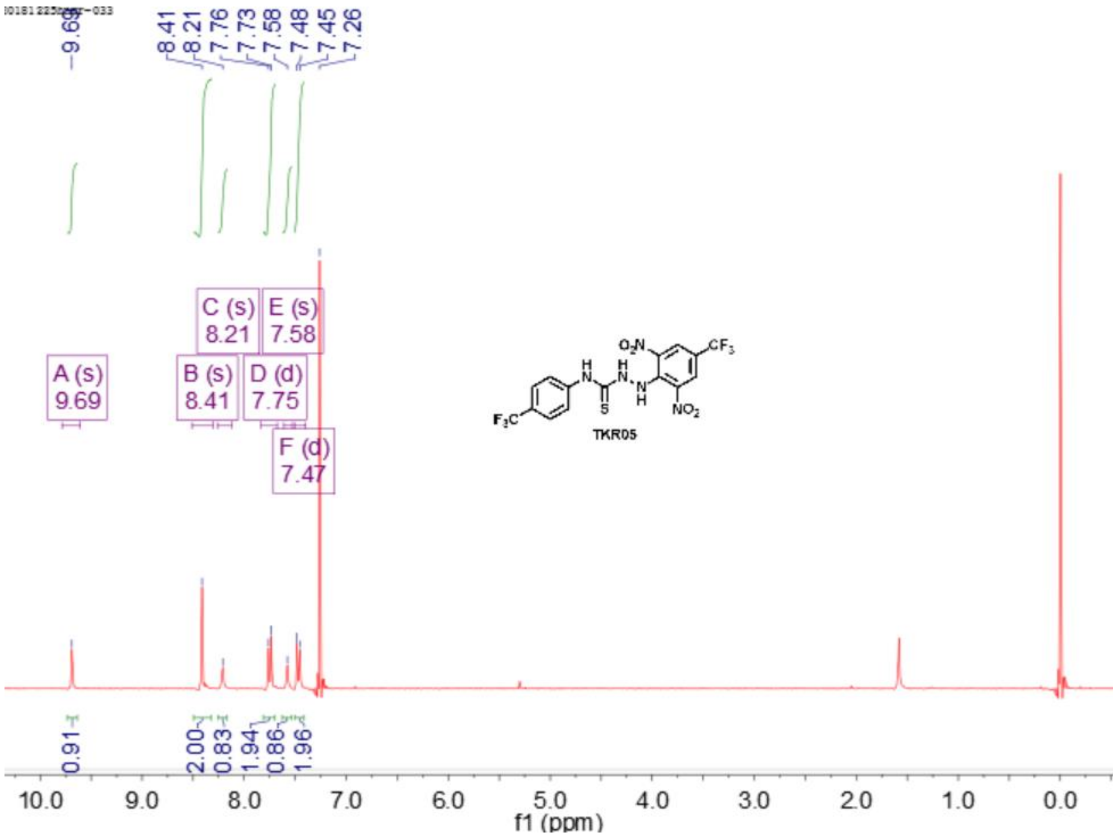
9.69 8.41 8.21 7.76 7.73 7.58 7.48 7.45 7.26
A (s) 9.69
B (s) 8.41
C (s) 8.21
D (d) 7.75
E (s) 7.58
F (d) 7.47
TKR05
0.91 2.00 0.83 1.94 0.86 1.96
10.0 9.0 8.0 7.0 6.0 5.0 4.0 3.0 2.0 1.0 0.0
f1 (ppm)

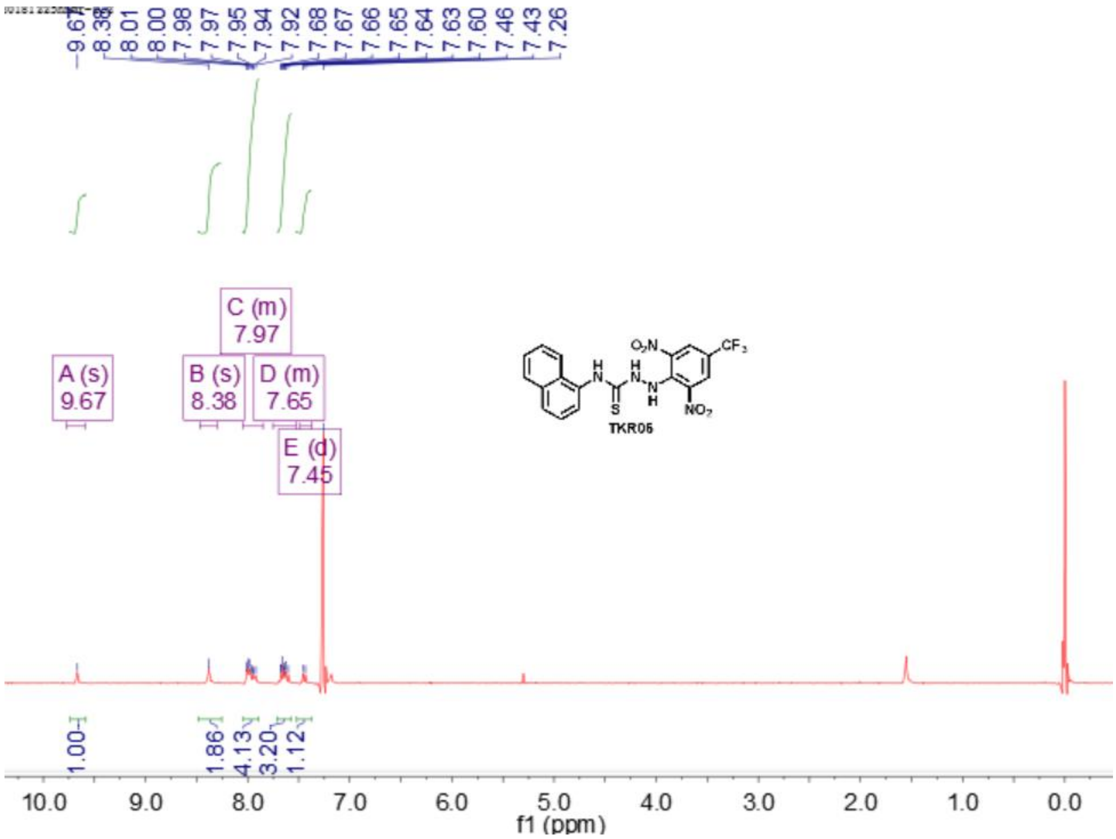

9.67 8.38 8.01 8.00 7.98 7.97 7.95 7.94 7.92 7.68 7.67 7.66 7.65 7.64 7.63 7.60 7.46 7.43 7.26
A (s) 9.67
B (s) 8.38
C (m) 7.97
D (m) 7.65
E (d) 7.45
TKR06
1.00 1.86 4.13 3.20 1.12
10.0 9.0 8.0 7.0 6.0 5.0 4.0 3.0 2.0 1.0 0.0
f1 (ppm)

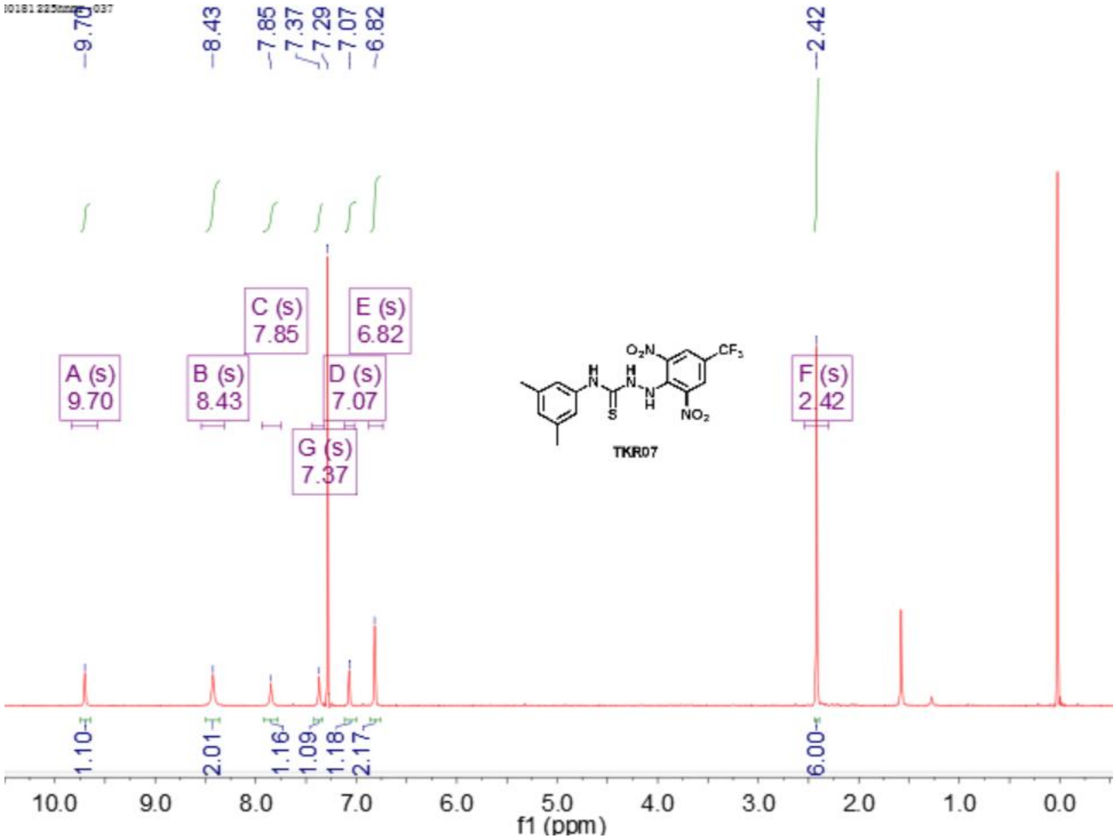

9.70 8.43 7.85 7.37 7.29 7.07 6.82 2.42
A (s) 9.70
B (s) 8.43
C (s) 7.85
D (s) 7.07
E (s) 6.82
F (s) 2.42
G (s) 7.37
TKR07
1.10 2.01 1.16 1.09 1.18 2.17 6.00
10.0 9.0 8.0 7.0 6.0 5.0 4.0 3.0 2.0 1.0 0.0
f1 (ppm)

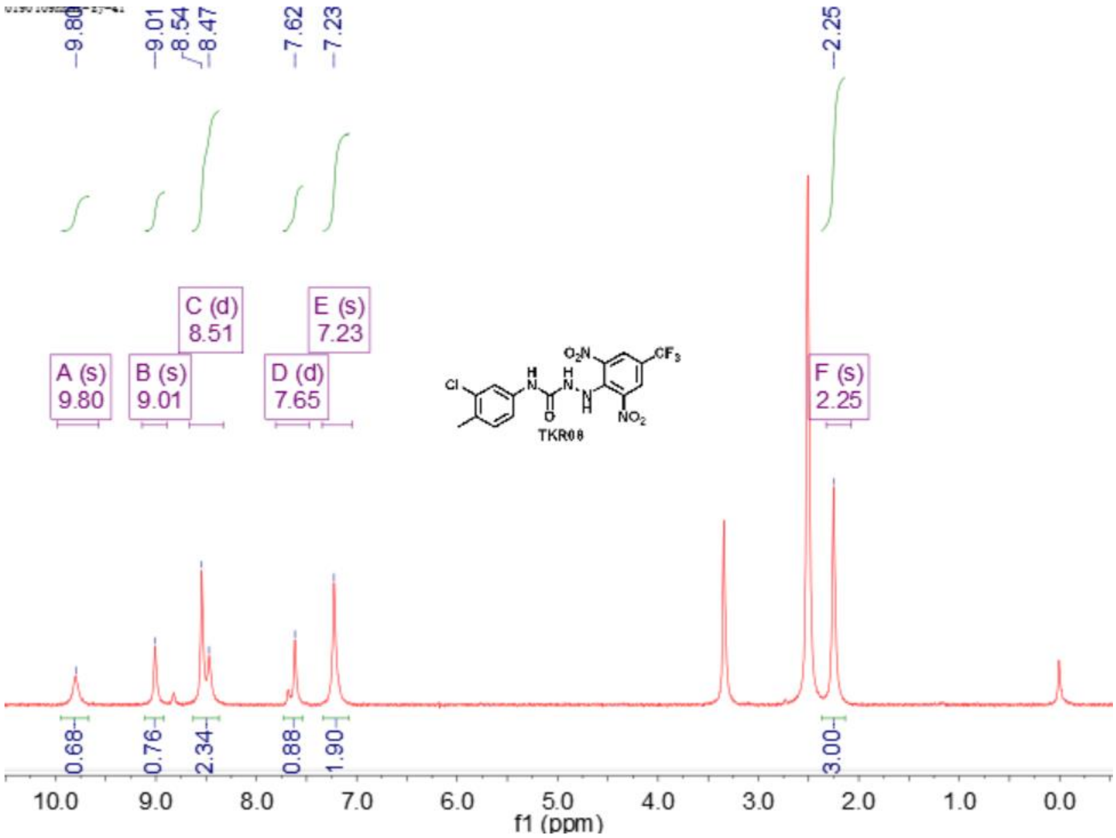
9.80
9.01
8.54
8.47
7.62
7.23
2.25
C (d) 8.51
E (s) 7.23
A (s) 9.80
B (s) 9.01
D (d) 7.65
F (s) 2.25
TKR08
0.68
0.76
2.34
0.88
1.90
3.00
10.0 9.0 8.0 7.0 6.0 5.0 4.0 3.0 2.0 1.0 0.0
f1 (ppm)

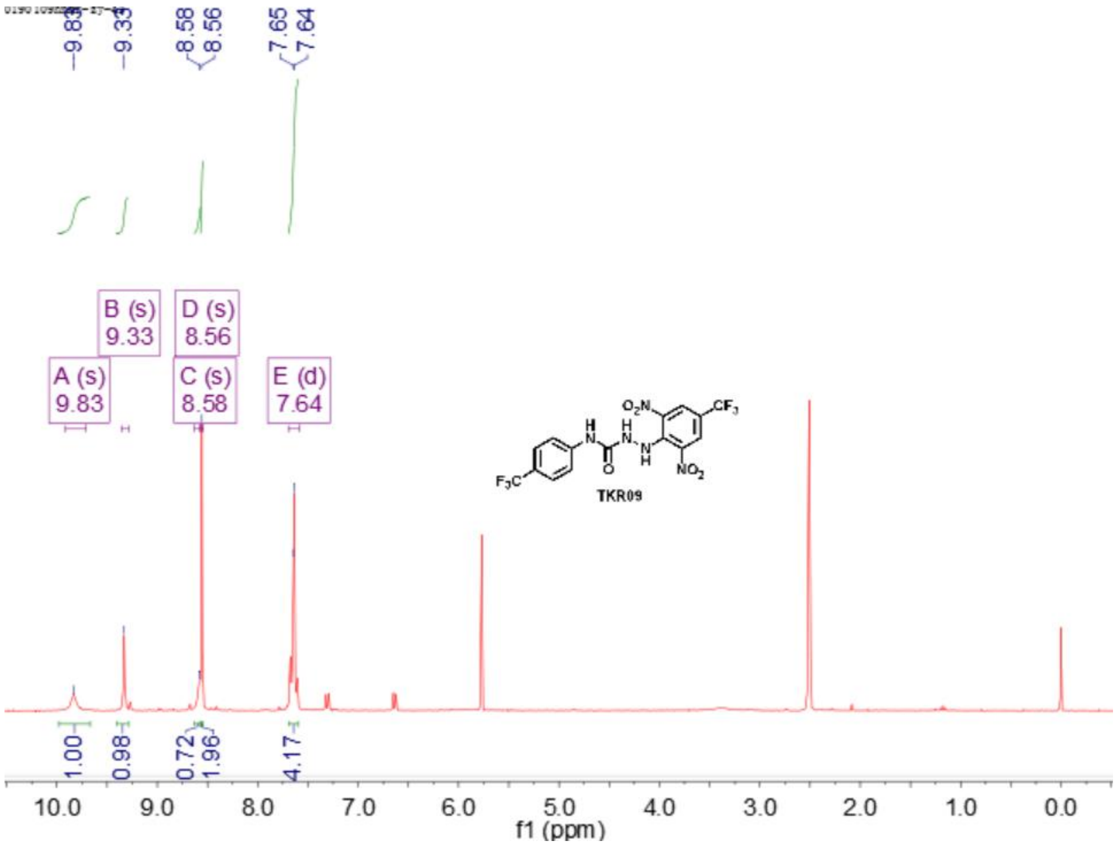
9.83
9.33
8.58
8.56
7.65
7.64
B (s) 9.33
D (s) 8.56
A (s) 9.83
C (s) 8.58
E (d) 7.64
TKR09
1.00
0.98
0.72
1.96
4.17
10.0 9.0 8.0 7.0 6.0 5.0 4.0 3.0 2.0 1.0 0.0
f1 (ppm)

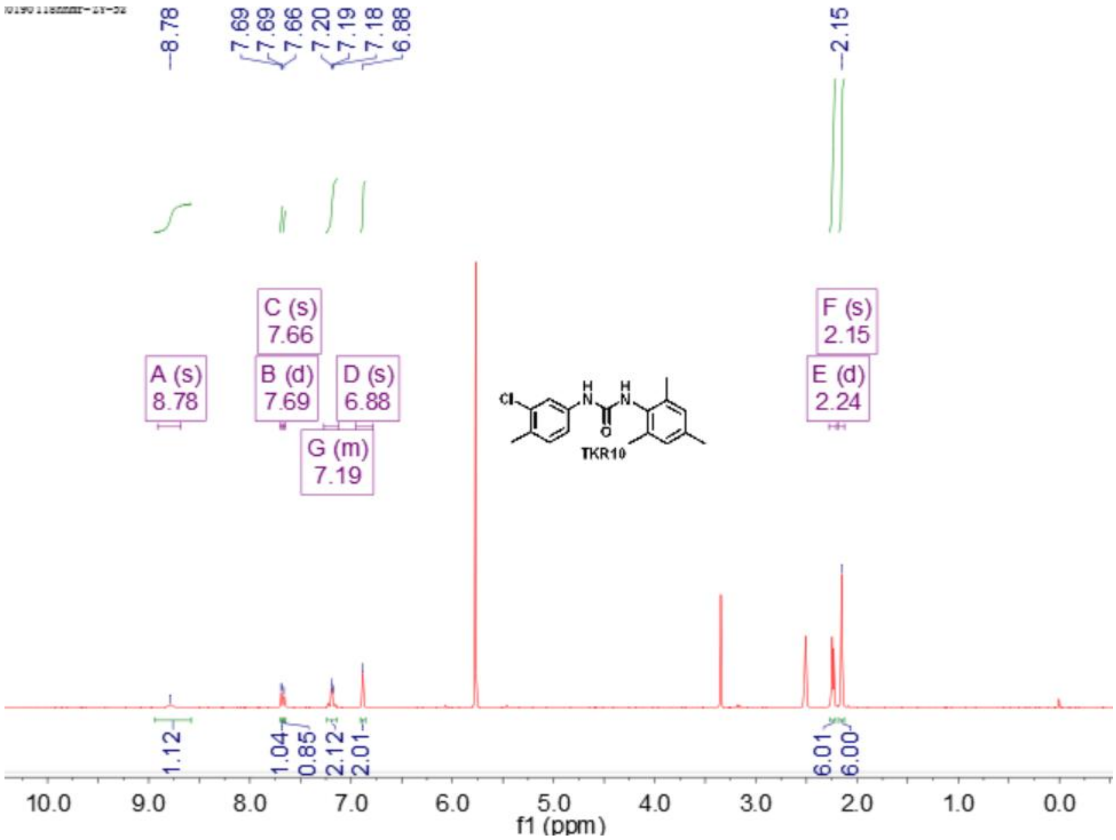
8.78
7.69
7.69
7.66
7.20
7.19
7.18
6.88
2.15
C (s) 7.66
A (s) 8.78
B (d) 7.69
D (s) 6.88
G (m) 7.19
F (s) 2.15
E (d) 2.24
Cl
H
N
O
TKR10
1.12
1.04
0.85
2.12
2.01
6.01
6.00
10.0
9.0
8.0
7.0
6.0
5.0
4.0
3.0
2.0
1.0
0.0
f1 (ppm)

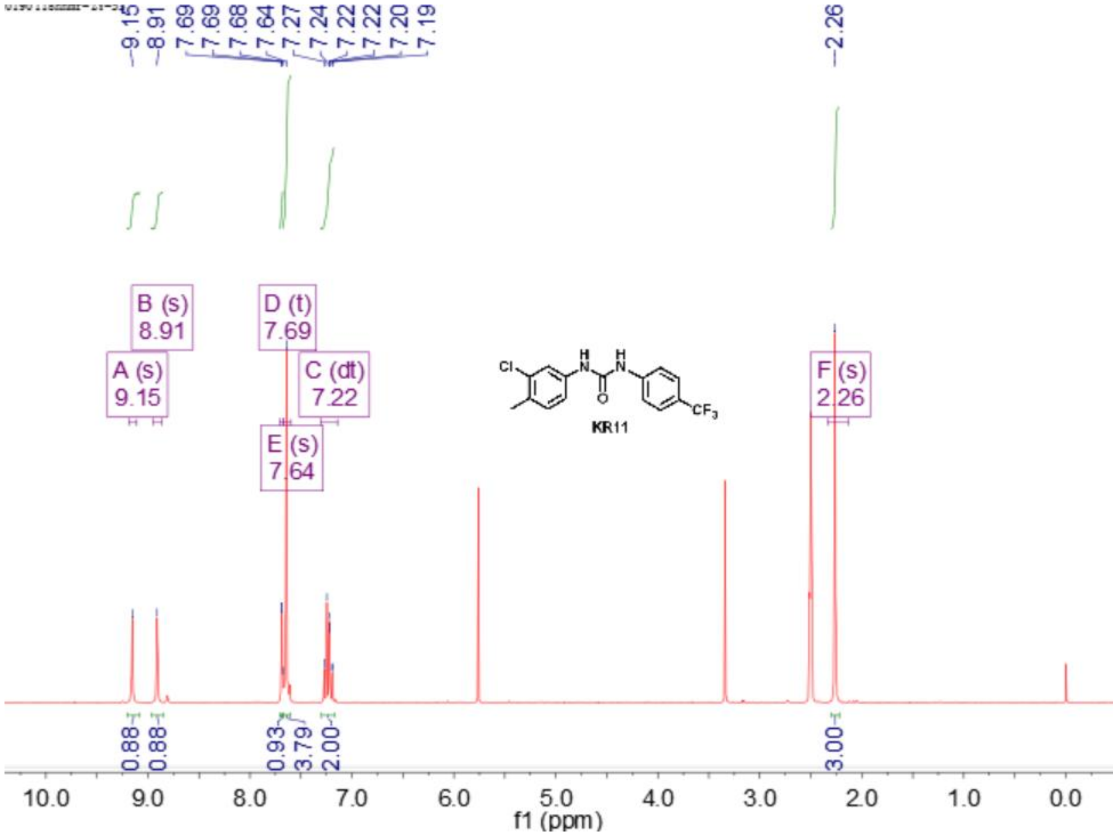
9.15
8.91
7.69
7.69
7.68
7.64
7.27
7.24
7.22
7.22
7.20
7.19
2.26
B (s) 8.91
D (t) 7.69
A (s) 9.15
C (dt) 7.22
E (s) 7.64
F (s) 2.26
Cl
H
N
O
CF_3
KR11
0.88
0.88
0.93
3.79
2.00
3.00
10.0
9.0
8.0
7.0
6.0
5.0
4.0
3.0
2.0
1.0
0.0
f1 (ppm)

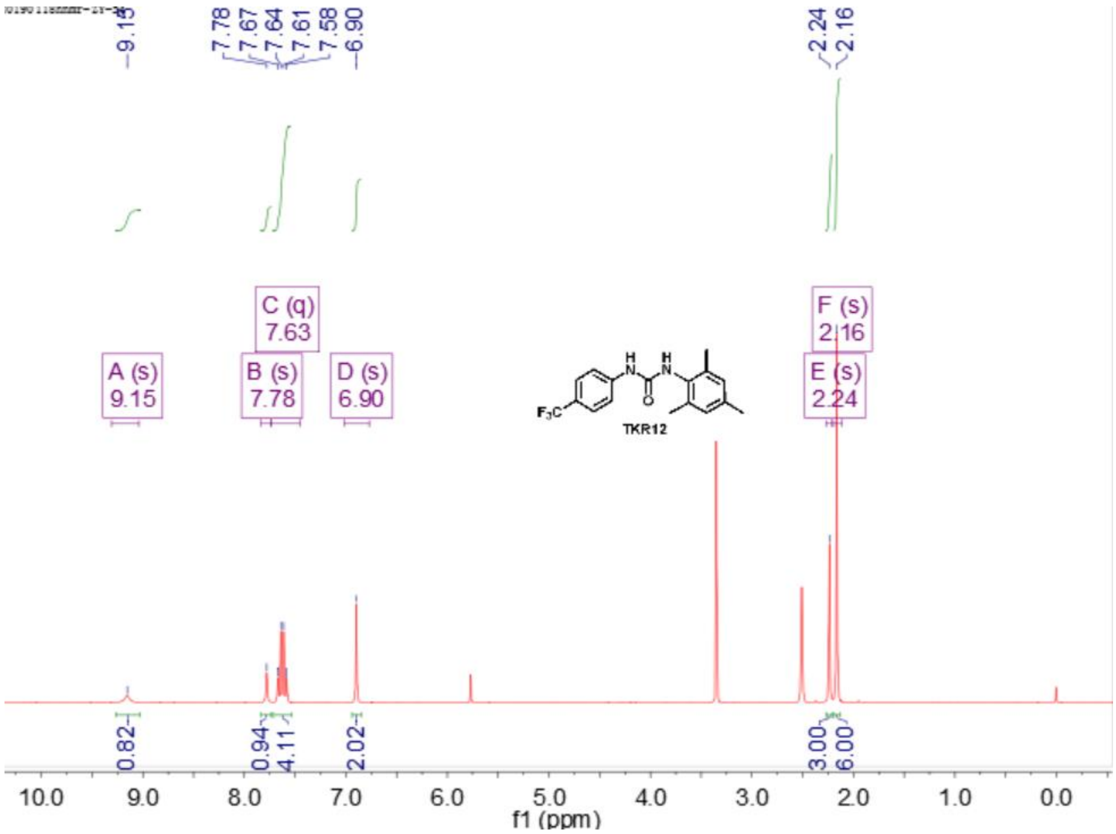

-9.15
7.78
7.67
7.64
7.61
7.58
-6.90
2.24
2.16
C (q)
7.63
A (s)
9.15
B (s)
7.78
D (s)
6.90
F (s)
2.16
E (s)
2.24
F_3C
TKR12
0.82
0.94
4.11
2.02
3.00
6.00
10.0 9.0 8.0 7.0 6.0 5.0 4.0 3.0 2.0 1.0 0.0
f1 (ppm)

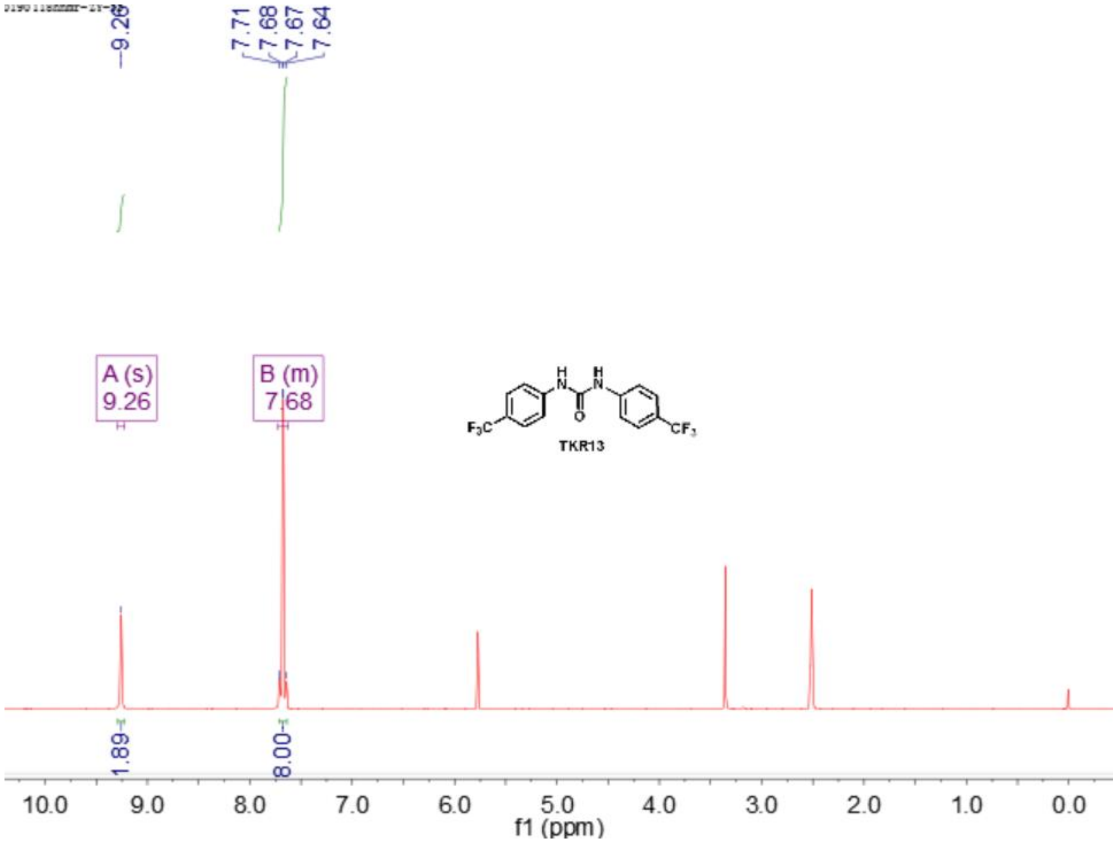

-9.26
7.71
7.68
7.67
7.64
A (s)
9.26
B (m)
7.68
F_3C
CF_3
TKR13
1.89
8.00
10.0 9.0 8.0 7.0 6.0 5.0 4.0 3.0 2.0 1.0 0.0
f1 (ppm)

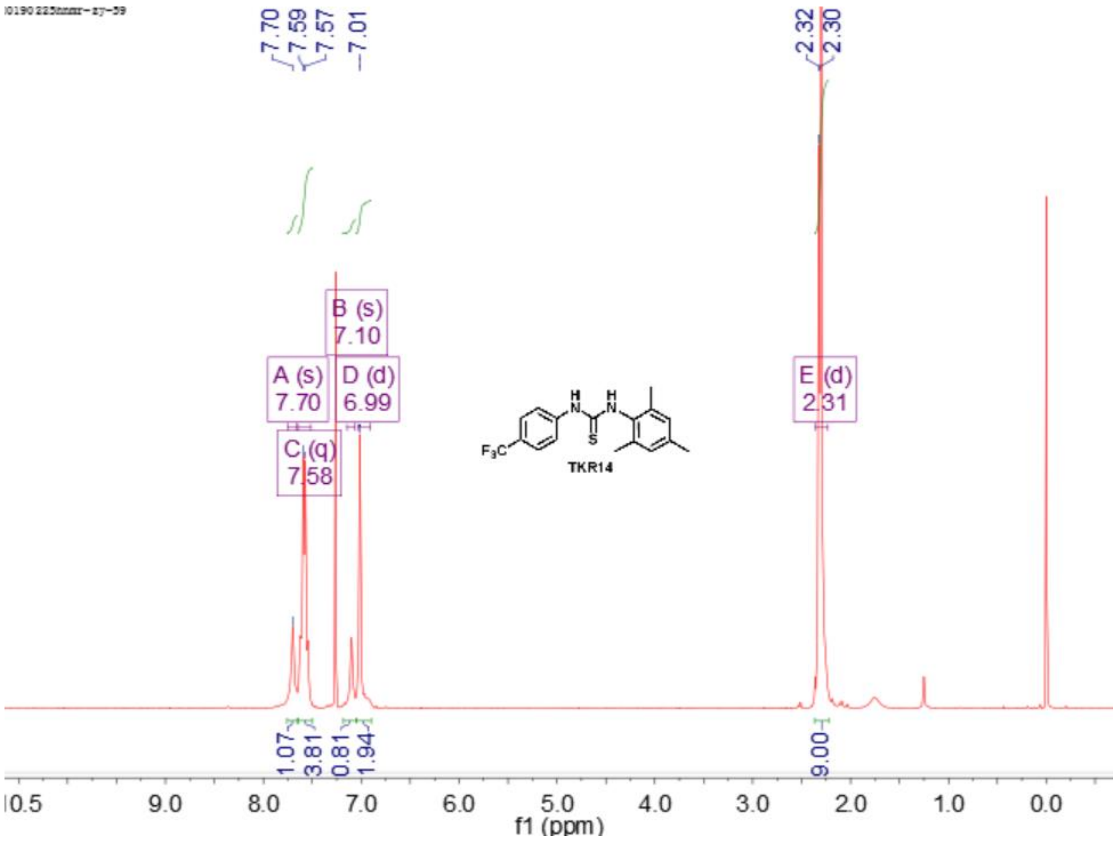
7.70
7.59
7.57
7.01
2.32
2.30
B (s)
7.10
A (s)
7.70
D (d)
6.99
C (q)
7.58
E (d)
2.31
TKR14
1.07
3.81
0.81
1.94
9.00
10.5
9.0
8.0
7.0
6.0
5.0
4.0
3.0
2.0
1.0
0.0
f1 (ppm)

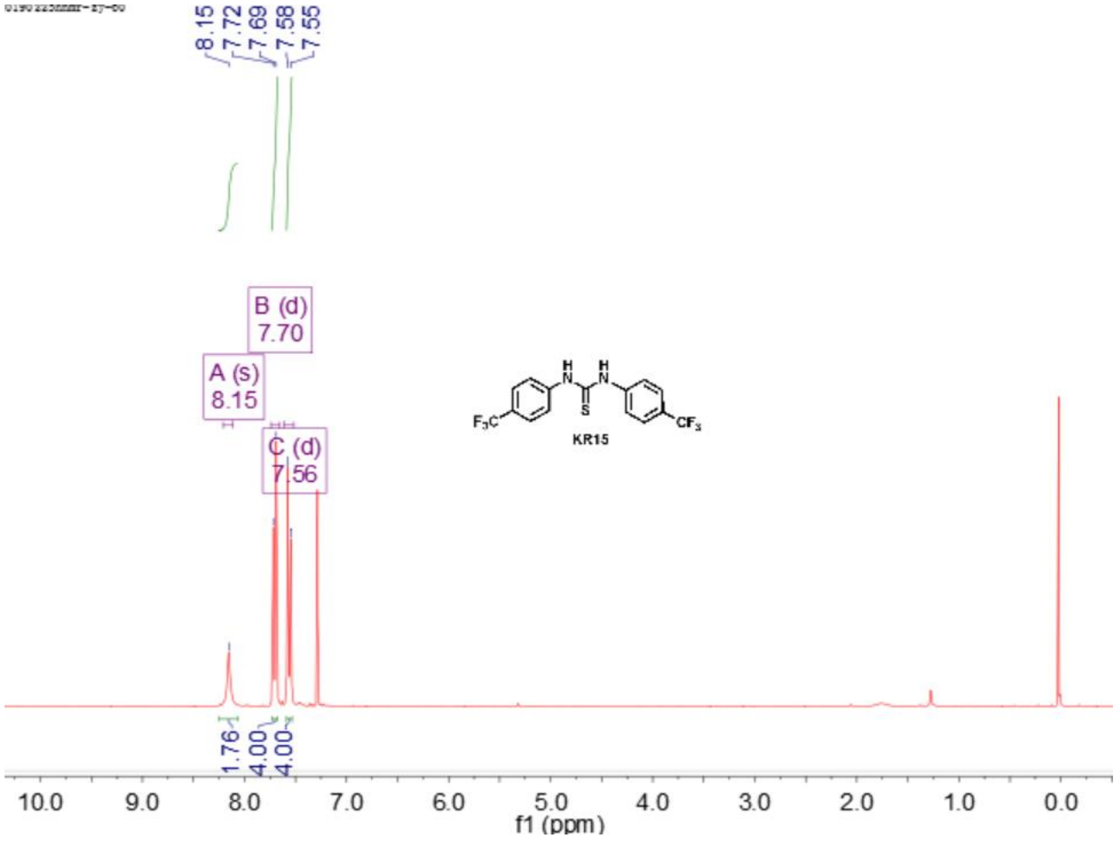
8.15
7.72
7.69
7.58
7.55
B (d)
7.70
A (s)
8.15
C (d)
7.56
KR15
1.76
4.00
4.00
10.0
9.0
8.0
7.0
6.0
5.0
4.0
3.0
2.0
1.0
0.0
f1 (ppm)

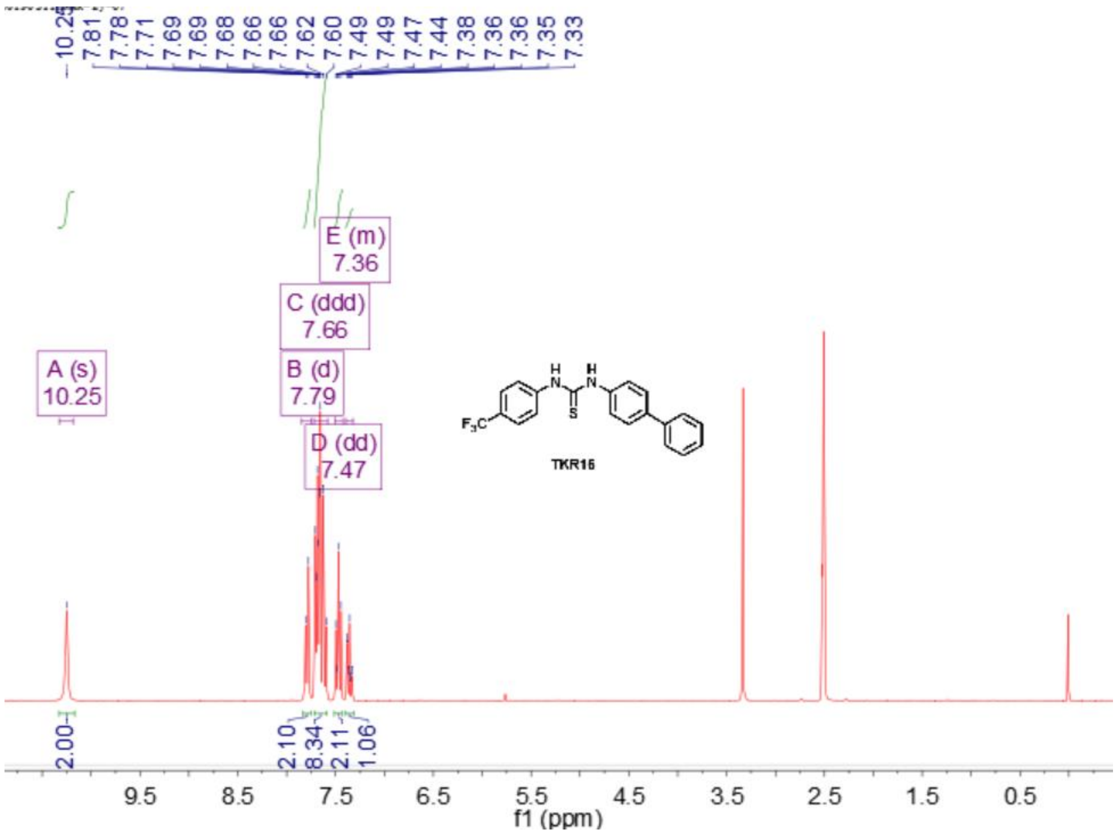
10.25
7.81
7.78
7.71
7.69
7.69
7.68
7.66
7.66
7.62
7.60
7.49
7.49
7.47
7.44
7.38
7.36
7.36
7.35
7.33
E (m) 7.36
C (ddd) 7.66
A (s) 10.25
B (d) 7.79
D (dd) 7.47
TKR15
2.00
2.10
8.34
2.11
1.06
9.5
8.5
7.5
6.5
5.5
4.5
3.5
2.5
1.5
0.5
f1 (ppm)

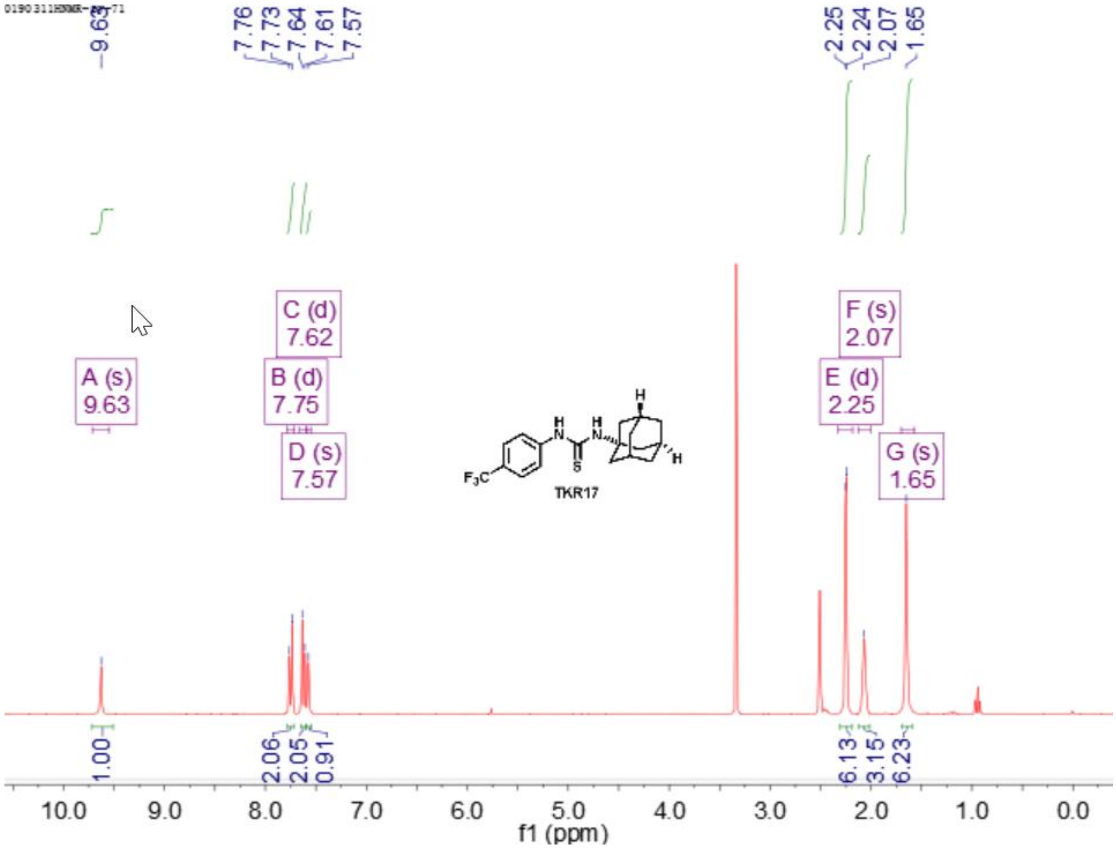
9.63
7.76
7.73
7.64
7.61
7.57
2.25
2.24
2.07
1.65
C (d) 7.62
A (s) 9.63
B (d) 7.75
D (s) 7.57
F (s) 2.07
E (d) 2.25
G (s) 1.65
TKR17
1.00
2.06
2.05
0.91
6.13
3.15
6.23
10.0
9.0
8.0
7.0
6.0
5.0
4.0
3.0
2.0
1.0
0.0
f1 (ppm)

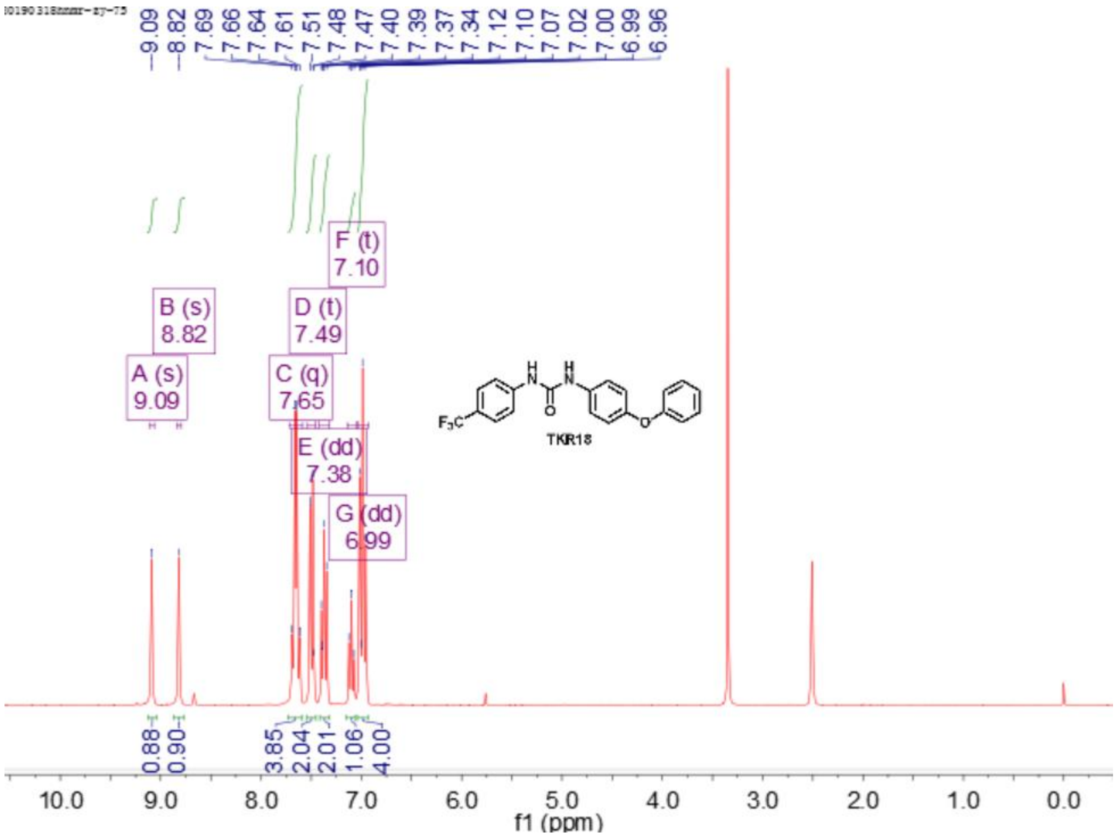

9.09 8.82 7.69 7.66 7.64 7.61 7.51 7.48 7.47 7.40 7.39 7.37 7.34 7.12 7.10 7.07 7.02 7.00 6.99 6.96
F (t) 7.10
B (s) 8.82
D (t) 7.49
A (s) 9.09
C (q) 7.65
E (dd) 7.38
G (dd) 6.99
F_3C
TKR18
0.88 0.90 3.85 2.04 2.01 1.06 4.00
10.0 9.0 8.0 7.0 6.0 5.0 4.0 3.0 2.0 1.0 0.0
f1 (ppm)

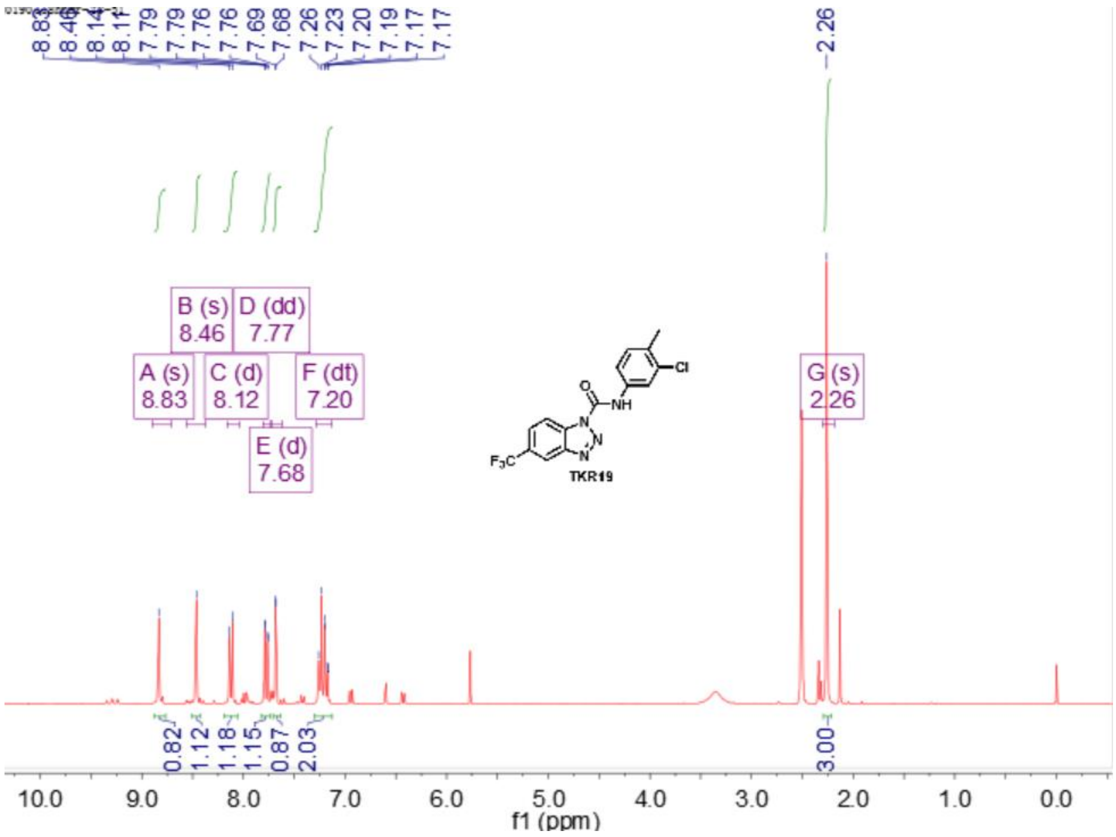

8.83 8.46 8.14 8.11 7.79 7.79 7.76 7.76 7.69 7.68 7.26 7.23 7.20 7.19 7.17 7.17
2.26
B (s) 8.46
D (dd) 7.77
A (s) 8.83
C (d) 8.12
F (dt) 7.20
E (d) 7.68
G (s) 2.26
Cl
NH
F_3C
TKR19
0.82 1.12 1.18 1.15 0.87 2.03
3.00
10.0 9.0 8.0 7.0 6.0 5.0 4.0 3.0 2.0 1.0 0.0
f1 (ppm)

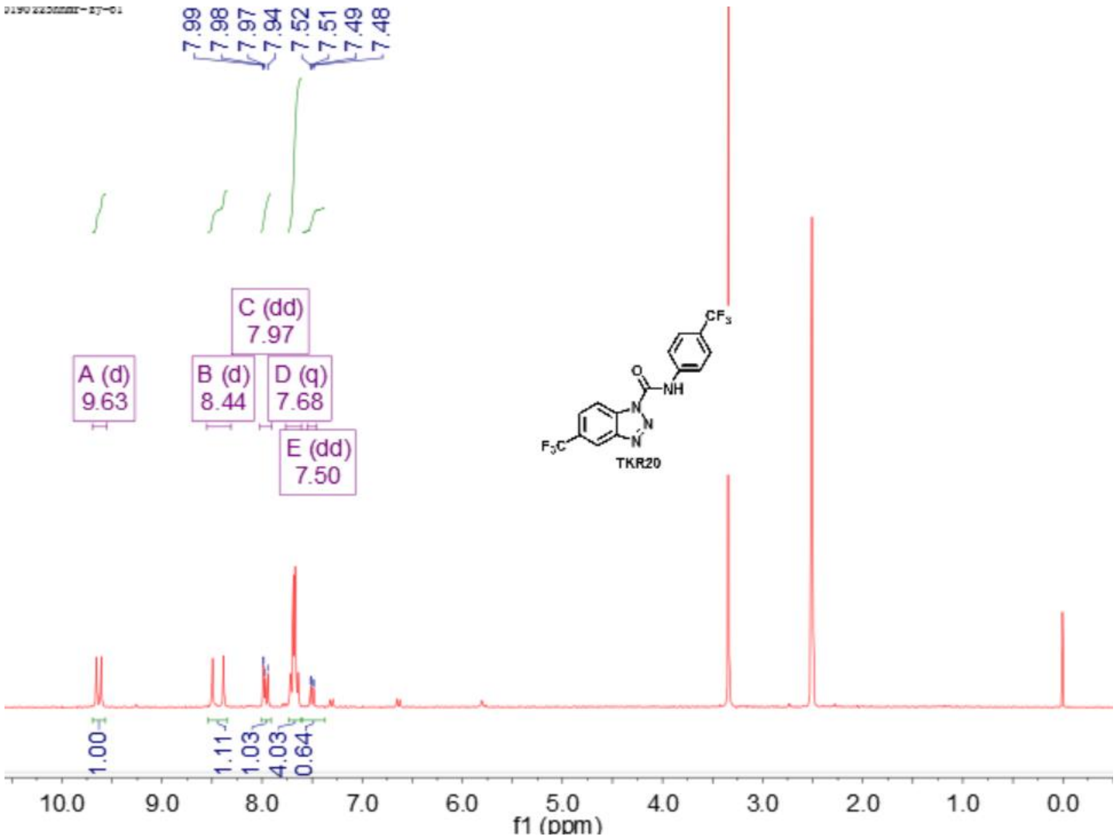

7.99
7.98
7.97
7.94
7.52
7.51
7.49
7.48
C (dd)
7.97
A (d)
9.63
B (d)
8.44
D (q)
7.68
E (dd)
7.50
CF3
O
NH
N
N
N
F3C
TKR20
1.00
1.11
1.03
4.03
0.64
10.0
9.0
8.0
7.0
6.0
5.0
4.0
3.0
2.0
1.0
0.0
f1 (ppm)

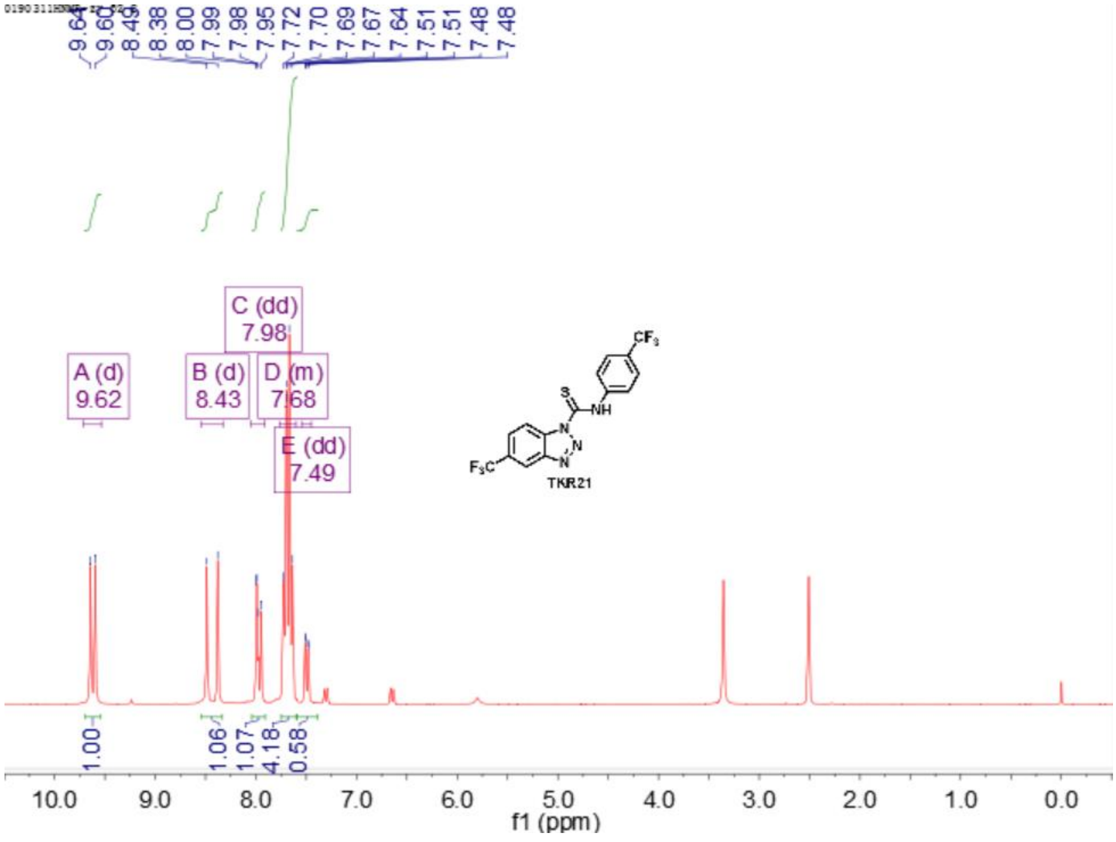

9.64
9.60
8.38
8.00
7.99
7.98
7.95
7.72
7.70
7.69
7.67
7.64
7.51
7.51
7.48
7.48
C (dd)
7.98
A (d)
9.62
B (d)
8.43
D (m)
7.68
E (dd)
7.49
CF3
S
NH
N
N
N
F3C
TKR21
1.00
1.06
1.07
4.18
0.58
10.0
9.0
8.0
7.0
6.0
5.0
4.0
3.0
2.0
1.0
0.0
f1 (ppm)
